# Amino acid transporter (AAT) gene family in foxtail millet (*Setaria italica* L.): widespread family expansion, functional differentiation, roles in quality formation and response to abiotic stresses

**DOI:** 10.1186/s12864-021-07779-9

**Published:** 2021-07-08

**Authors:** Yang Yang, Yongmao Chai, Jiayi Liu, Jie Zheng, Zhangchen Zhao, Aduragbemi Amo, Chunge Cui, Qiumei Lu, Liang Chen, Yin-Gang Hu

**Affiliations:** 1 State Key Laboratory of Crop Stress Biology for Arid Areas, College of Agronomy, Northwest A&F University, Yangling, Shaanxi China; 2 Institute of Water Saving Agriculture in Arid Regions of China, Northwest A&F University, Yangling, Shaanxi China

**Keywords:** Foxtail millet, Amino acid transporter, Genome-wide characterization, Functional differentiation, Grain quality, Abiotic stresses

## Abstract

**Background:**

Amino acid transporters (AATs) plays an essential roles in growth and development of plants, including amino acids long-range transport, seed germination, quality formation, responsiveness to pathogenic bacteria and abiotic stress by modulating the transmembrane transfer of amino acids. In this study, we performed a genome-wide screening to analyze the *AAT* genes in foxtail millet (*Setaria italica* L.), especially those associated with quality formation and abiotic stresses response.

**Results:**

A total number of 94 *AAT* genes were identified and divided into 12 subfamilies by their sequence characteristics and phylogenetic relationship. A large number (58/94, 62%) of *AAT* genes in foxtail millet were expanded via gene duplication, involving 13 tandem and 12 segmental duplication events. Tandemly duplicated genes had a significant impact on their functional differentiation via sequence variation, structural variation and expression variation. Further comparison in multiple species showed that in addition to paralogous genes, the expression variations of the orthologous *AAT* genes also contributed to their functional differentiation. The transcriptomic comparison of two millet cultivars verified the direct contribution of the AAT genes such as *SiAAP1*, *SiAAP8*, and *SiAUX2* in the formation of grain quality. In addition, the qRT-PCR analysis suggested that several *AAT* genes continuously responded to diverse abiotic stresses, such as *SiATLb1*, *SiANT1*. Finally, combined with the previous studies and analysis on sequence characteristics and expression patterns of *AAT* genes, the possible functions of the foxtail millet *AAT* genes were predicted.

**Conclusion:**

This study for the first time reported the evolutionary features, functional differentiation, roles in the quality formation and response to abiotic stresses of foxtail millet *AAT* gene family, thus providing a framework for further functional analysis of *SiAAT* genes, and also contributing to the applications of *AAT* genes in improving the quality and resistance to abiotic stresses of foxtail millet, and other cereal crops.

**Supplementary Information:**

The online version contains supplementary material available at 10.1186/s12864-021-07779-9.

## Background

Amino acids, as small-molecule organic compounds containing nitrogen, are the main carriers of nitrogen exchange in plants and the nutrients necessary for plant growth, development and quality [1]. The absorption and transport of amino acids are mainly mediated by amino acid transporters (AATs) [2]. Through the transmembrane transport of amino acids, amino acid transporters play essential roles in plant growth and development, including seed germination [3, 4], long-distance transport of amino acids [5], quality formation [6, 7], and response to pathogenic bacteria [8] and abiotic stress [9]. A total of 72, 65, 189, 85 and 297 amino acid transporters have been characterized in potato [10], *Arabidopsis* [1], soybean [11], rice [12] and wheat [13], respectively, suggesting that amino acid transporters are widely present in higher plants.

The *AAT* gene family in plants belongs to APC transporter superfamily, that comprises of two main families of amino acid/auxin permease (AAAP) family and amino acid-polyamine-choline (APC) transporter family. The AAAP family consists of Amino acid permeases (AAPs), lysine and histidine transporters (LHTs), auxin transporters (AUXs), c-aminobutyric acid transporters (GATs), neutral amino acid transporters (ANTs), proline transporters (ProTs), and amino acid transporter-like a/b (ATLa/b) proteins [8, 14, 15]. The APC family comprises cationic amino acid transporters (CATs), amino acid/choline transporters (ACTs), polyamine H^+^ − symporters (PHSs), and tyrosine-specific transporters (TTPs) subfamilies [10, 16].

Based on the heterologous expression system, the functions of *AAT* genes in *Arabidopsi*s have been characterized in detail [17]. There are 8 *AAP* genes (*AtAAP1*-*AtAAP8*), and 6 *AtAAP* genes transport charged and neutral amino acids with various affinities [17, 18]. In endosperm, roots and cotyledon, AtAAP1 is abundantly expressed, which is essential for the synthesis of storage proteins in *Arabidopsis* seed and seed yield, while regulating embryonic development and amino acid input in roots [14, 19]. *AtAAP2* and *AtAAP3* are mainly involved in the xylem-phloem transfer of amino acids and the absorption of amino acids in xylem, respectively [20, 21]. *AtAAP4* and *AtAAP5* can transfer amino acids to developing embryos and *AtAAP5* plays crucial role in the root amino acid intake system [22]. *AtAAP6* affects the interaction between plants and aphids through the regulation of the phloem’s amino acid composition [8]. *AtAAP8* is mainly responsible for transporting amino acids into the endosperm and embryo at the early embryonic development [23]. Additionally, the functions of some *AAPs* in other species have also been explored. In *Vicia faba*, *VfAAP1* is expressed abundantly in cotyledons and other pool tissues, and significantly affects the storage protein content (GPC) in seeds by regulating the nitrogen content of seeds [4, 6], while *VfAAP3* is significantly enriched in roots, stems, pods, pistils and seed coats at various stages of development [4]. In potato, *StAAP1* is expressed during the source-sink transition process, and the free amino acid contents are decreased in transgenic plants with reduced *StAAP1* expression [24], and the expression patterns of *StAAP4*, 5, and 6 may be associated with the long-distance transport of amino acids [10]. In barley, *HvAAP2* and *6* are mainly expressed in nutritive organs, while *HvAAP3* is high-expressed in seeds and contributes significantly to nitrogen accumulation in developing seed [25]. In rice, *OsAAP1* affects plant growth and grain yield by modulating the redistribution of neutral amino acids [26], and the quantitative trait locus qPC1 (QTL) for modulation of rice GPC is linked to the expression of *OsAAP6* [7]. In addition, several wheat *TaAAP* genes have been confirmed to co-localize with QTL of grain protein content [27].

In addition to the in-depth studies on *AAP* genes, the functions of some genes in other subfamilies have also been explored [5, 8, 9]. *AtLHT1* plays an indispensable role in the uptake of amino acids and mesophyll formation in root, while *AtLHT2* mainly involves in the movements and allocation of amino acids in floral organs [28–30]. *AtANT1* is expressed abundantly in flowers and stem leaves, transporting neutral and aromatic amino acids as well as indole-3-acetic acid (IAA) and arginine [31]. *AtAUX1* and *AtLAX3* affect root gravitropism and lateral root formation by facilitating IAA sink-source distribution and auxin-induced expression of root cell-wall-remodeling genes, respectively [32–35]. *AtProT1* and *AtProT2* participate in the long-distance transportation of proline and the absorption of proline and glycine betaine in roots of *Arabidopsis*, respectively [36, 37]. *AtProT3* is mainly expressed in leaf epidermal cells, which may be involved in the regulation of proline distribution in leaves [38]. AtCAT5 mediates the reabsorption of amino acids permeated at leaf margin by transporting basic amino acids, while AtCAT3, AtCAT6 and AtCAT8 prefer to transport acidic or neutral amino acids [17]. AtBAT1 has been identified as a bidirectional amino acid transporter expressed on mitochondrial membrane, which mediates γ-aminobutyric acid (GABA) from cytosol to mitochondria [39].

In addition to the role of *AAT* genes in seed germination, long-distance amino acid transport, root gravitropism, the formation of lateral roots and reproductive organs, a number of AAT genes play an important role in reducing damage to plants under various abiotic stresses, specifically by promoting the transportation of stress-response compounds and compatible solutes such as proline, GABA, and betaine [40]. For example, the *ProT* genes rapidly distribute proline to minimize damage to plants caused by water shortage [41]. *AtProT2* is strongly induced by salt stress, and plays a role in nitrogen distribution under salt stress [42]. *GmProT1* and *GmProT2* are sensitive to stress in the roots and transgenic plants can transport more proline after spraying proline [43]. *AtGAT1* is significantly induced by GABA enrichment in plants under mechanical damage, stress or sensitive environment [44]. *OsAAP6*, *OsAAP11* and *OsANT3* are strongly up-regulated under salt and drought stresses, to regulate the tolerance to stress of plants through the transport of stress compounds [12].

Foxtail millet (*Setaria italica* L.), a member of the Poaceae family, is one of the oldest cereal crops, domesticated in Northern China. It is rich in essential amino acids, fatty acids and minerals, which is of important significance to human health [45]. To date, the *AAT* gene family in foxtail millet has not been characterized. Given their essential roles in the growth and grain quality formation of foxtail millet, we carried out a detailed characterization of *AAT* genes in foxtail millet. The aims of this study are to: (a) Accurately identify and characterize *AAT* genes in foxtail millet based on the latest genome sequence; (b) Explore the roles of paralogous and orthologous *AAT* genes in the build-up and functional differentiation of AAT family in foxtail millet through comparative analysis of their gene structure, sequence feature and expression patterns; (c) Mine the key candidate *AAT* genes that affect grain quality formation of foxtail millet through the comparative transcriptome analysis of two genotypes; (d) Detect the response of *SiAAT* genes to diverse abiotic stresses by Quantitative Real-Time PCR; (e) Predict the possible functions of the *SiAAT* genes based on the previous studies, sequence composition and expression pattern. This work will provide a deeper insight and understanding of the *AAT* genes in foxtail millet. Also, important clues for their functional analysis and applications in improving quality and resistances to abiotic stresses will be provided.

## Results

### Identification of AAT gene family in foxtail millet

Firstly, 104 putative *AAT* transcripts were identified in foxtail millet based on local BLASP. However, 94 high-confidence non-redundant *AAT* genes were confirmed after screening the conservative domains by HMMER, CDD and Interpro databases and removing the different transcripts of the same gene and sequences containing incomplete conserved domains, which was basically at the same level as other gramineous species besides hexaploid wheat (Table 1; Additional file 5: Table S1). Except for tetraploid soybean, *AAT* gene family in foxtail millet was expanded than that in *Arabidopsis* and potato. Overall, the number of *AAT* genes in monocots was higher than that in dicots. The *AAT* genes identified in foxtail millet were renamed according to their chromosomal localizations and phylogenetic relationships with other species. The length of AAT proteins in foxtail millet ranged from 311aa to 984aa, with molecular weight (Mw) varing from 4.97 kD to 107.5 kD, and isoelectric point (pI) ranged from 4.94 to 9.99 (Additional file 5: Table S1). Different subfamilies of *AAT* genes showed abundant diversity in subcellular localization, with the subfamily members of AAP, LHT, GAT, AUX and ProT all located on plasma membrane, while those of TTP, ACT, ANT and ATLb all located on vacuole membrane. Some members of the same subfamily showed different subcellular localization, such as, different genes of the ATLa subfamily located on both membranes of plasma and vacuole, while that of the CAT and PHS subfamilies located on the three membranes of the plasma, vacuole and chloroplast.

**Table 1:** Comparison on the gene abundance of twelve subfamilies of *AAT* genes in 6 monocots and 3 eudicots

	Monocots						Eudicots		
	Millet^**a**^	Sorghum	Maize	***Brachypodium***	Rice	Wheat	Soybean	***Arabidopsis***	Potato
AAAP									
AAP	20(21.27%)	17(22.07%)	24(22.43%)	19(23.75%)	19(22.35%)	66(22.30%)	35(18.52%)	8(12.70%)	8(11.11%)
LHT	12(12.77%)	8(10.39%)	15(14.42%)	8(10.00%)	6(7.05%)	24(8.11%)	24(12.70%)	10(15.87%)	11(15.28%)
GAT	6(6.38%)	4(5.19%)	2(1.92%)	3(3.75%)	4(4.71%)	14(4.73%)	19(10.05%)	2(3.17%)	3(4.17%)
ProT	1(1.06%)	1(1.30%)	2(1.92%)	2(2.50%)	3(3.53%)	9(3.04%)	7(3.70%)	3(4.76%)	4(6.35%)
AUX	4(4.25%)	5(6.49%)	6(5.60%)	3(3.75%)	5(5.88%)	15(5.07%)	16(8.47%)	4(6.35%)	5(7.94%)
ATLa	6(6.38%)	6(7.79%)	7(6.54%)	6(7.50%)	7(8.24%)	18(6.08%)	16(8.47%)	5(7.94%)	8(11.11%)
ANT	2(2.13%)	4(5.19%)	3(2.8%)	5(6.25%)	4(4.71%)	18(6.08%)	6(3.17%)	4(6.34%)	5(7.94%)
ATLb	14(14.89%)	8(10.39%)	17(15.89%)	7(8.75%)	10(11.76%)	40(13.51%)	30(15.87%)	10(15.87%)	8(11.11%)
APC									
ACT	8(8.51%)	5(6.49%)	7(6.54%)	6(7.50%)	7(8.24%)	21(7.09%)	7(3.70%)	1(1.59%)	1(1.39%)
CAT	12(12.77%)	11(14.29%)	14(13.08%)	11(13.75%)	11(12.94%)	31(10.47%)	19(10.05%)	9(14.29%)	9(12.5%)
PHS	8(8.51%)	7(9.09%)	7(6.54%)	7(8.75%)	9(10.59%)	31(10.47%)	9(4.76%)	5(7.94%)	8(11.11%)
TTP	1(1.06%)	1(1.30%)	3(2.80%)	3(3.75%)	0(0.00%)	9(3.04%)	1(0.53%)	2(3.17%)	2(2.78%)
Total	94	77	107	80	85	296	189	63	72

^a^The numbers represent the number of identified AAT subfamily members, and the percentages in the brackets represent the proportion to all *AAT* genes

**Fig. 1:**
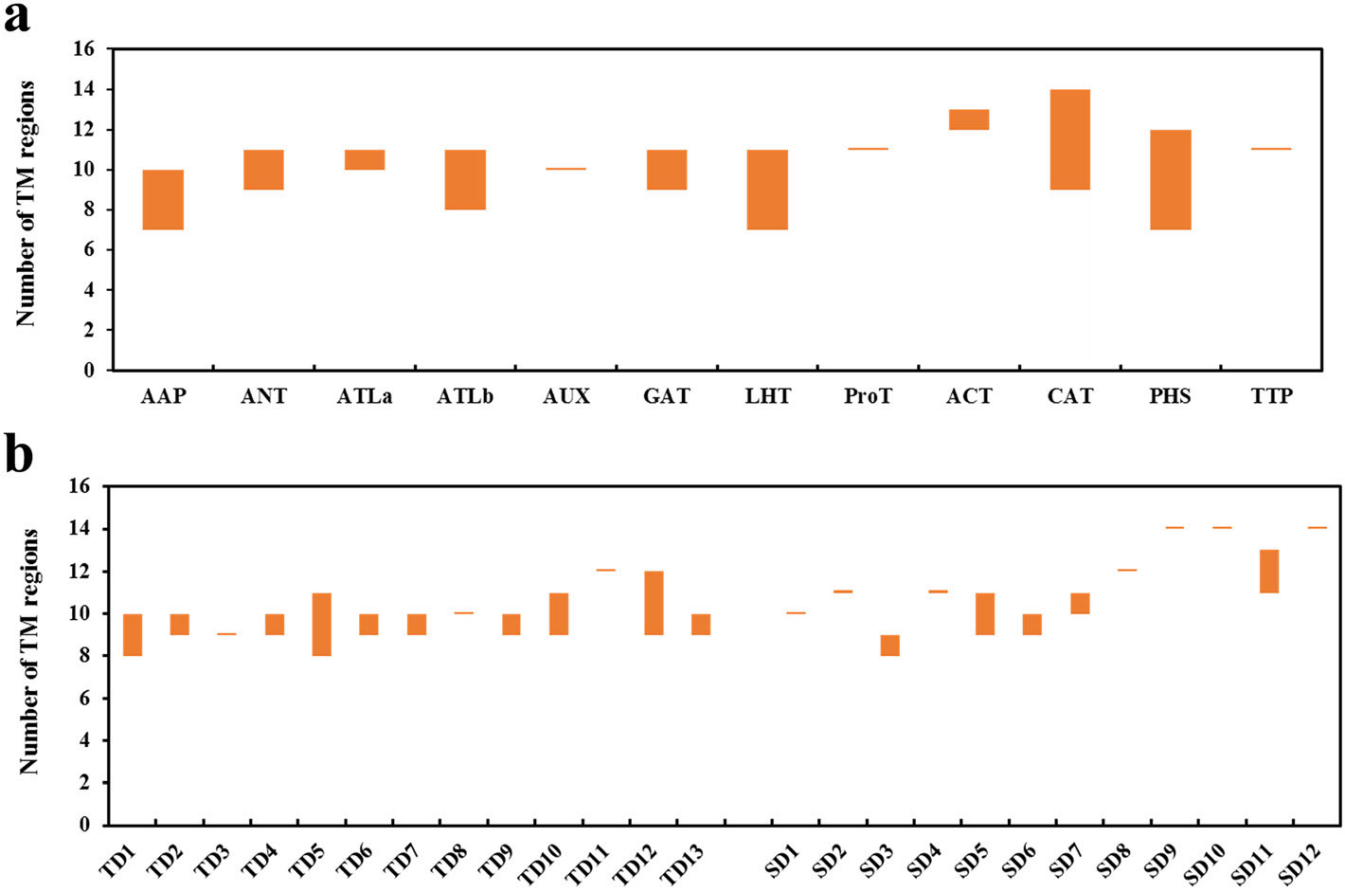
The variations in the number of putative transmembrane (TM) regions of AAT proteins in foxtail millet. (**a**) The X-axis and Y-axis represent the 12 AAT subfamily members and the number of TM regions, respectively; (**b**) The X-axis lists all duplicated gene groups, and Y-axis represents the number of TM regions

The number of predicted TM regions in SiAATs varied in different subfamilies, which ranged from 7 to 14 (Fig. 1a; Additional file 5: Table S1). For example, all AUXs contained 10 TM regions, ATLas contained 10 or 11 TM regions, while CAT and PHS subfamilies ranged from 9 to 14 and 7 to 12, respectively.

### Chromosomal distribution and duplication events of *AAT* genes in foxtail millet

Here, 93 of the 94 *SiAATs* were mapped to 9 chromosomes, the remaining one was mapped to an unassembled scafford, which were unevenly distributed, with 18 on chromosome 7 and 2 on chromosome 2 as the most and least, respectively (Additional file 1: Fig. S1). The apparent regional enrichment of *SiAATs* on some chromosomes was observed on their distributions. For instance, a large number of *SiAATs* were located on the ends of chromosomes 1, 5, and 7. Moreover, some *SiAATs* were mainly concentrated at the fronts of chromosomes 6 and 8 (Additional file 1: Fig. S1).

Of the 94 *AAT* genes in foxtail millet, 58 (62%) were involved in gene duplication events, including 36 tandem and 25 segmental duplications, of which *SiATLb12*, *SiLHT2* and *SiLHT9* were involved in both duplications (Additional file 1: Fig. S1; Additional file 5: Table S1). The 36 tandem duplications were classified into 13 groups, of which 2 groups with 5 genes (TD10, TD11), 1 group with 4 genes (TD3), 2 groups with 3 genes (TD1, TD13), and the remaining 8 groups all with 2 genes (Additional file 5: Table S1). The 25 segmental duplications were classified into 12 groups, except for SD3 containing 3 genes, with other possessing 2 genes. Gene duplication events produced a large number of paralogous AAT genes, thus promoting the significant build-up of the *AAT* gene family in foxtail millet.

### The phylogenetic tree and structure of *AAT* genes in foxtail millet

**Fig. 2:**
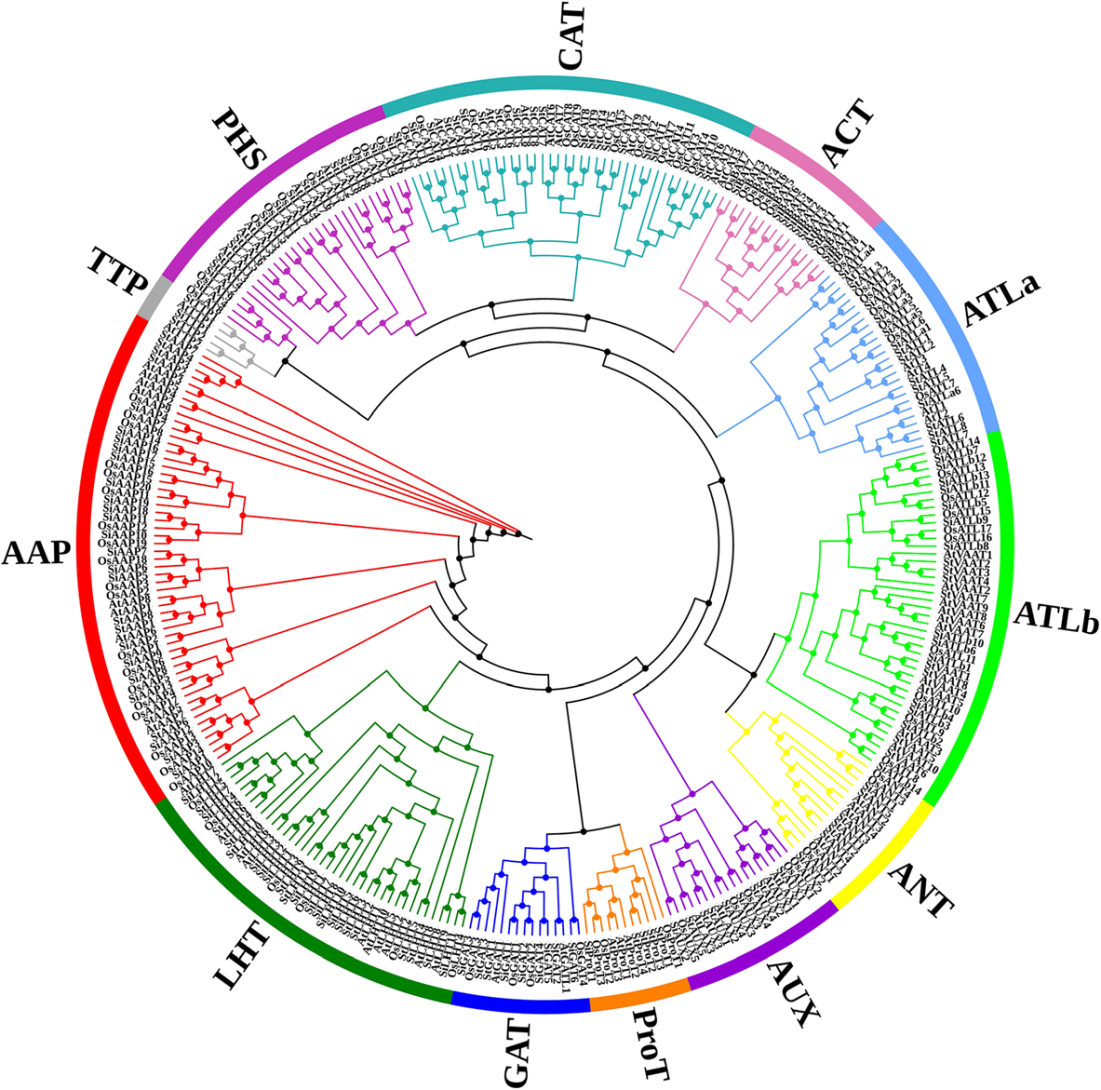
Phylogenetic tree constructed by the AAT proteins of foxtail millet, rice, *Arabidopsis* and potato. MUSCLE and MEGA 6.06 were used to perform the multiple sequence alignment and to construct the phylogenetic tree with the maximum likelihood (ML) method and the JTT model. Different AAT subfamily members were shown with different colored branches. AAT proteins were named according to the ID in published articles, such as OsAAP1, AtAAP1 and StAAP1

The phylogenetic analysis with the AAT protein sequences from foxtail millet (94), rice (85), *Arabidopsis* (63) and potato (72) showed that they could be divided into twelve genetic groups with high confidence and that from Monocots and Eudicots distributed in the same group (Fig. 2). The 65 SiAAT proteins of the AAAP family were divided into 8 subfamilies of amino acid permeases (AAPs, 20), lysine, histidine transporters (LHTs, 12), GABA transporters (GATs), proline transporters (ProTs, 1), aux transporters (AUXs, 4), amino acid transporter-like a (ATLa, 6), aromatic and neutral amino acid transporters (ANTs, 2) and amino acid transporter-like b (ATLb, 14). The 29 SiAAT proteins of the APC family were clearly distinguished into 4 subfamilies of cationic amino acid transporters (CATs, 12), amino acid/choline transporters (ACTs, 8), tyrosine-specific transporters (TTPs, 1), and polyamine H^+^-symporters (PHSs, 8) (Fig. 2).

**Fig. 3:**
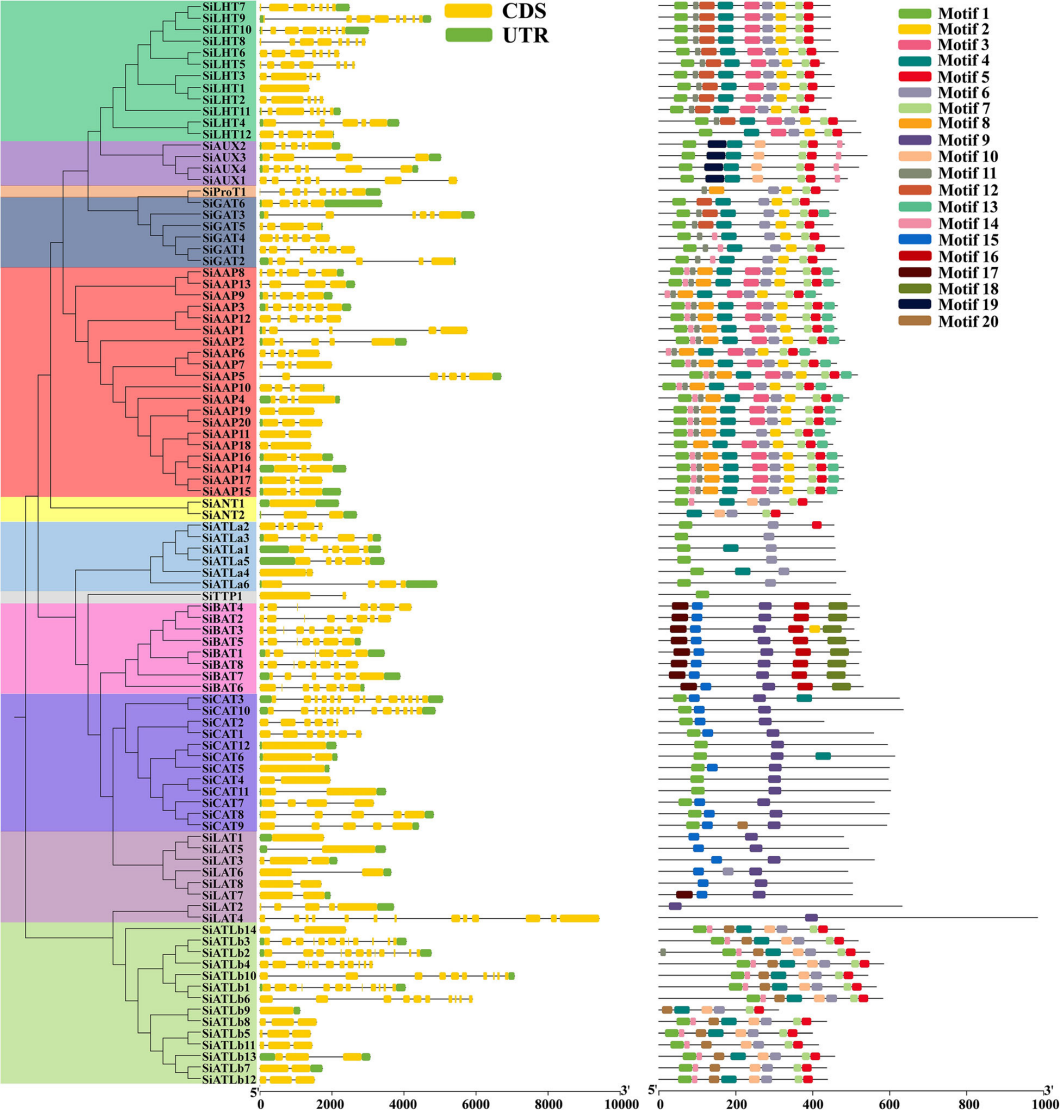
Gene structure and conserved motifs of *SiAATs* in each subfamily. Different subfamilies of foxtail millet AAT family are shown with various color backgrounds. The gene structure was referred from the gene model file of foxtail millet reference genome. The exons, UTR regions and introns are displayed by green boxes, yellow boxes and the block lines, respectively. The detail motif sequences are as listed in Additional file 2: Fig. S2

Based on their annotation information, similar exon/intron structures were observed in most of the *SiAATs* in the same subfamily, such as *SiATLa1* and *SiATLa5*, *SiCAT3* and *SiCAT10*, and *SiATLb3* and *SiATLb2.* Variants were also found in some *SiAATs* of the same subfamily, such as *SiAUX2* and *SiAUX3*, *SiAAP11* and *SiAAP18*, and *SiANT1* and *SiANT2* (Fig. 3). The conserved motifs of SiAAT proteins predicted with MEME were highly consistent with the phylogenetic relationship and classification of SiAATs (Fig. 3; Additional file 2: Fig. S2). Similar to their gene structures, the presence of the conserved motifs in different subfamilies of SiAAT proteins varied. For instance¸Motif 1, 4, and 7 were widespread in the AAAP family, while Motif 9 existed in the APC family. Some motifs were unique to certain subfamilies, for example, AUX subfamily only possessed Motif 19, and Motif 16 and 18 were only found in the ACT subfamily.

### Variations in gene structure, conserved domains and sequences of paralogous *SiAAT* genes

The diversification of paralogous gene is one of the important sources of the functional differentiation of gene families [46–48]. The comparison on the TM regions of SiAATs found that significant variations in the number of TM regions occurred in the same duplicated gene groups, 60% of the duplicated gene groups (15/25) varied in the number of TM regions, which was 77% (10/13) and 42% (5/12) for the tandemly and for the segmentally duplicated gene groups, respectively (Fig. 1b; Table 2).

**Table 2:** The extensive variations in gene structure, protein structure and sequence of paralogous *AAT* genes produced by gene duplication in foxtail millet

Type of duplication	Subfamily	No. of duplicated gene groups	Gene and protein structure variation			Range of Ka/Ks values	Duplicated gene groups
			No. of TM variation	No. of gene structure variation	No. of conserved domain variation		
Tandem duplication	AAP	4	3	3	2	0.12–0.62	TD1-TD4
	ATLb	3	3	1	2	0.27–0.47	TD5-TD7
	GAT	1	0	1	1	0.43	TD8
	LHT	2	2	2	0	0.10–0.39	TD9,TD10
	ACT	1	0	1	1	0.18–0.27	TD11
	CAT	1	1	1	0	0.97	TD12
	PHS	1	1	0	1	0.41–0.67	TD13
	Sum^a^	13	10 (77%)	9 (69%)	7 (54%)	0.10–0.97 (0.28)	
Segmental duplication	ATLa	2	0	0	2	0.10–0.12	SD1,SD2
	ATLb	3	2	2	2	0.12–0.40	SD3-SD5
	LHT	2	2	1	0	0.12–0.37	SD6,SD7
	ACT	1	0	1	0	0.10	SD8
	CAT	4	1	1	3	0.14–0.50	SD9-SD12
	Sum^a^	12	5 (42%)	5 (42%)	7 (58%)	0.10–0.50 (0.21)	

^a^The data in columns 4, 5 and 6 represents the total number and its proportion (in the brackets) of mutated duplicated gene groups, while that in column 7 represents the range and average (in the brackets) of Ka/Ks values

**Fig. 4:**
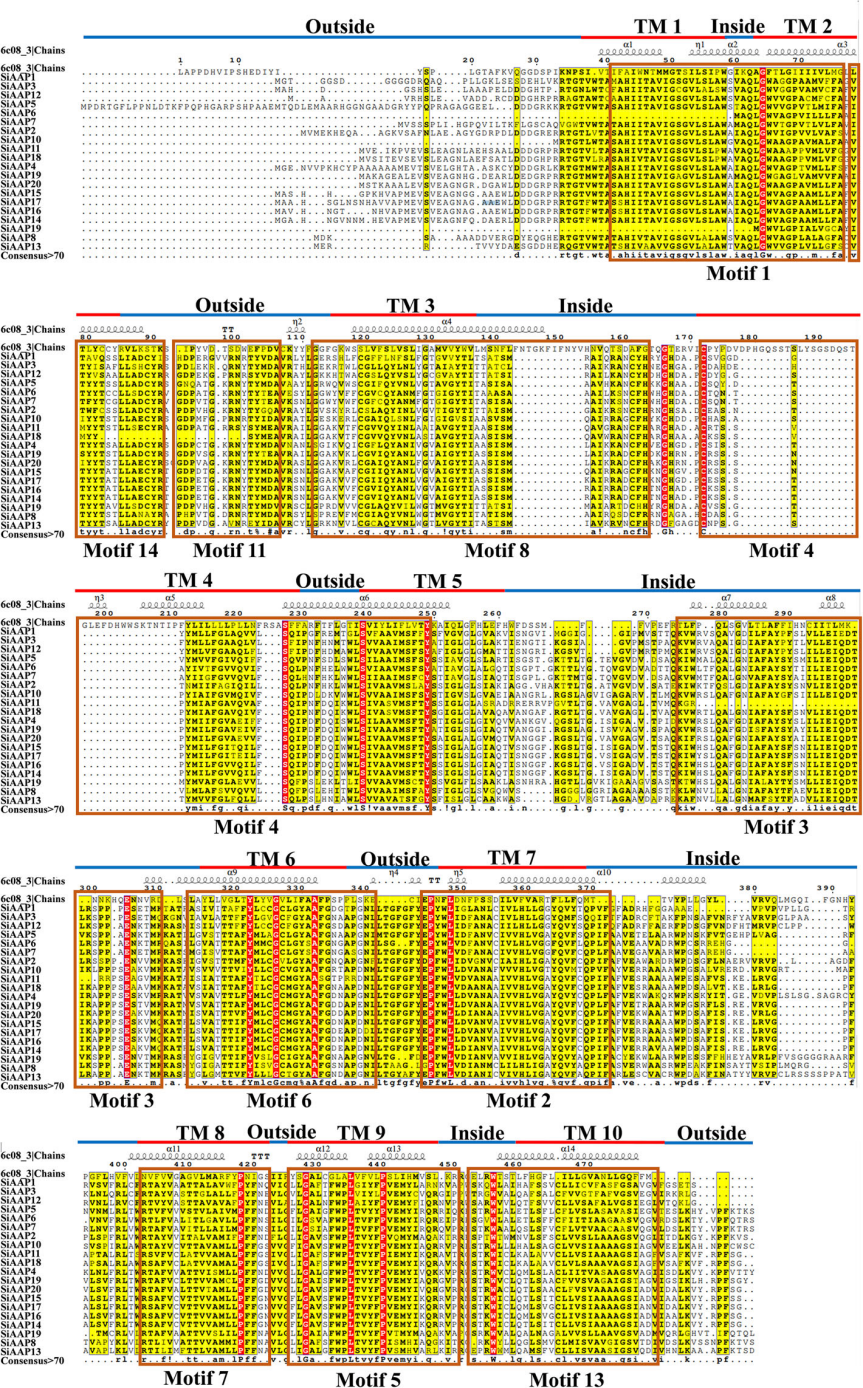
The multiple-sequence alignment of the AAP proteins in foxtail millet. The TM regions of SiAAP proteins are indicated with red line, and their conserved motifs predicted by MEME are shown in the red boxes

Multi-sequence alignment of SiAAP proteins revealed that their overall similarity was 59.65% with 11 conserved motifs, and their TM regions were highly correlated with the conserved motifs, both in length and amino acid composition (Fig. 4). Motif 1 and 14 both formed the TM 1 and TM 2 regions, TM 4 and part of TM 5 regions included Motif 4. TM regions of 3, 6, 7, 8, 9 and 10 were with Motifs of 8, 6, 2, 7, 5, 13, respectively (Fig. 4). In addition, some conserved motifs were located in the non TM region, such as Motif 11 in the extra-membrane region and Motif 3 in the intra-membrane region (Fig. 4). It was worth noting that some SiAAPs had missing transmembrane regions due to incomplete conserved motifs, such as SiAAP6 and SiAAP9. Therefore, the number variation of TM regions was mainly determined by the presence of different conserved domains. Prediction of the secondary structure of SiAAT proteins found 14 α-helixes and 4 η-helixes structures, with vast majority located in the TM regions to ensure the efficient and stable transmembrane transport of amino acids (Fig. 4).

In terms of gene structure, 56% (14/25) of duplicated gene groups had variation in the number of introns, which was 69% (9/13) of the tandemly ones and 42% of the segmentally ones (Table 2; Additional file 3: Fig. S3). In addition, the variations in the conserved motifs were also observed in 14 duplicated gene groups, 7 for each of the two types of duplication (Table 2).

**Fig. 5:**
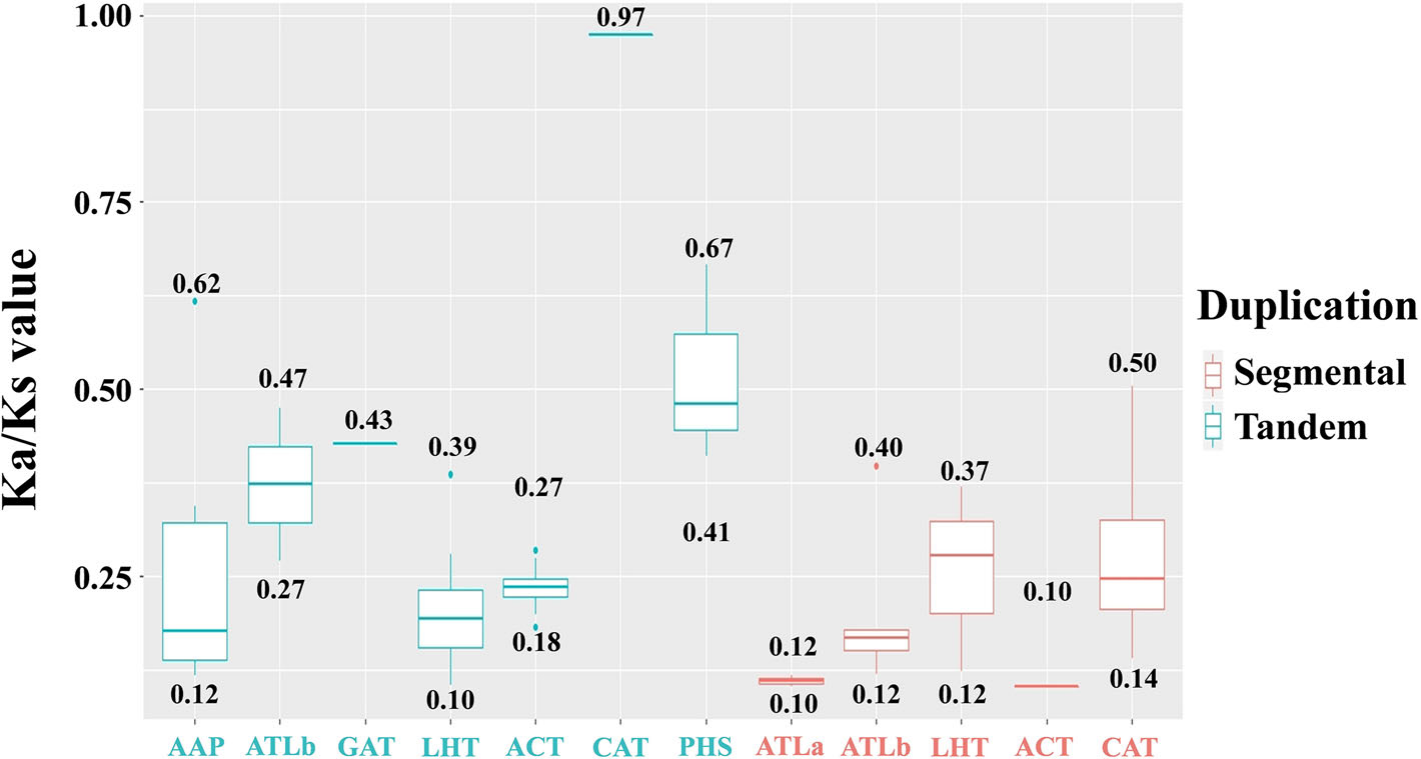
The Ka/Ks values of homologous *AAT* gene groups in different AAT subfamilies of foxtail millet. The X-axis and Y-axis indicates the duplicated *AAT* gene pairs in different subfamilies and Ka/Ks (nonsynonymous / synonymous) values, respectively. Segmental duplications and tandem duplications were shown in red and blue, respectively

The Ka/Ks values of all paralogous *SiAAT* gene pairs were less than 1, ranged from 0.1 to 0.97, which suggested that these genes were subject to different levels of purifying selection (Fig. 5). The tandemly duplicated gene pairs had higher Ka/Ks values than the segmental duplications, with the average Ka/Ks values of 0.28 and 0.21, respectively (Table 2). The Ka/Ks values of tandemly duplicated genes of PHS subfamily ranged from 0.41 to 0.67, while that of PHS subfamily only ranged from 0.18 to 0.27. The Ka/Ks values of different subfamilies also showed different degrees of dispersion. For instance, that of *SiAAPs* ranged from 0.12 to 0.62, while that of *SiBATs* (ACT subfamily) from 0.18 to 0.27. This similar phenomenon was also observed on the Ka/Ks values of segmentally duplicated genes (Fig. 5). In general, significant variations in gene structure, conserved domains and sequences among the paralogous *SiAAT* gene groups might greatly promote the functional diversity of the *AAT* gene family in foxtail millet.

### Variations in expression levels of paralogous and orthologous *SiAAT* genes

**Fig. 6:**
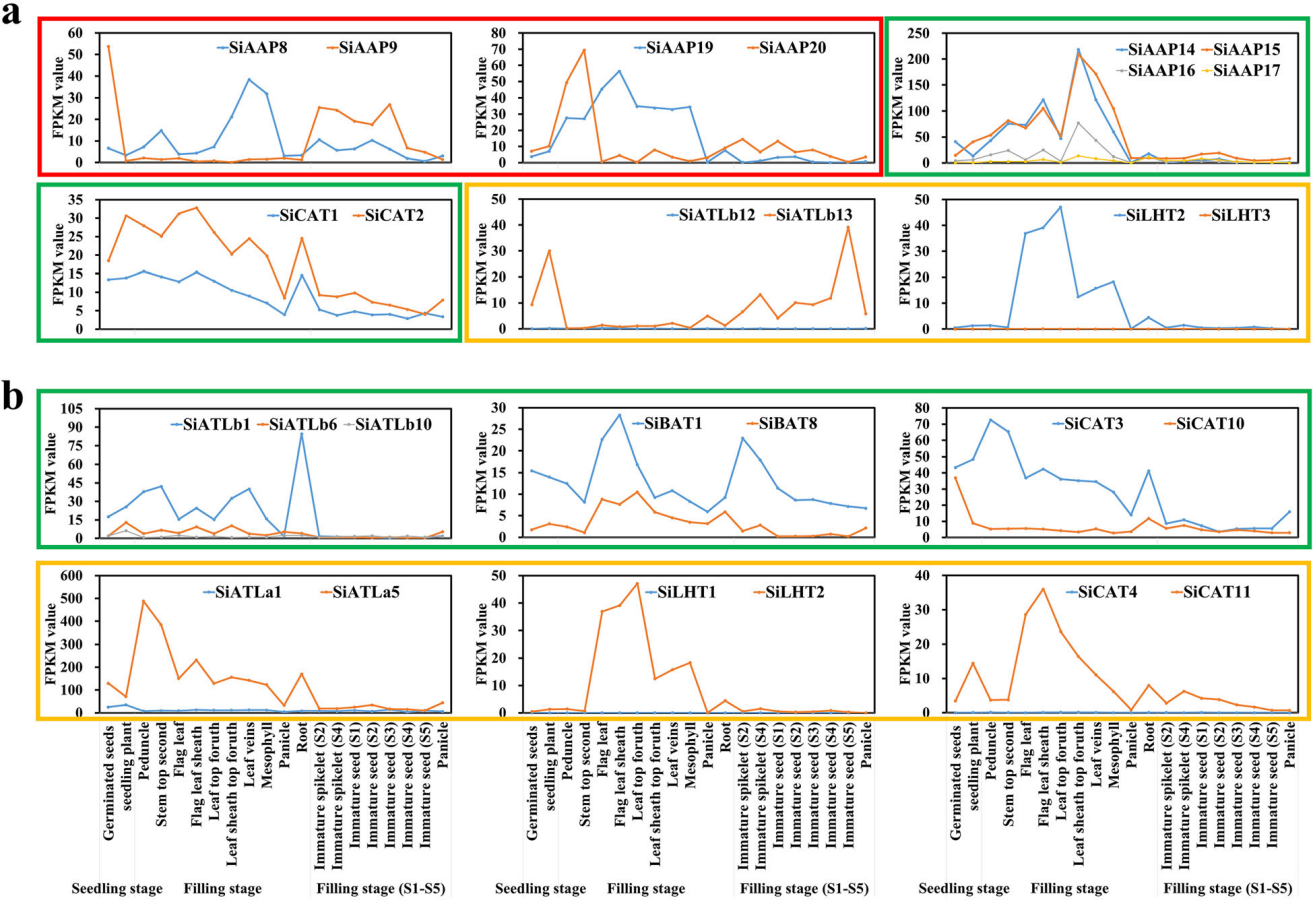
Three variations of expression patterns of duplicated *AAT* gene groups in foxtail millet. (**a**) and (**b**) is for the tandemly and segmentally duplicated gene groups, respectively. The X-axis indicates different tissue at different stage, and the Y-axis represents the FPKM value. Different colored boxes represent different expression patterns. Red boxes: new-functionalization; Blue boxes: sub-functionalization; Yellow boxes: non-functionalization

The pattern of expression of all paralogous *AAT* genes were investigated using the public transcriptome data. Compared with the ancestral genes, according to their expression patterns, the newly duplicated genes could be classified into three main types of new-functionalization, sub-functionalization and non-functionalization, which were all observed in the tandemly duplicated gene groups with *SiAAP8/9* (TD2) possessing new functions. *SiAAP9* and *SiAAP8* were highly expressed during grain development, and in leaves at the filling stage, respectively. *SiAAP14/15/16/17* (TD3) and *SiCAT1/2* (TD12) showed sub-functionalization, as the expressions of *SiAAP16/17* and *SiCAT2* were down-regulated significantly. In addition, *SiATLb12* and *SiLHT3* losing their functions, as they were not expressed in any tissues (Fig. 6a). Though no new function was observed, similar functional differentiation were also observed in the segmentally duplicated gene groups, such as sub-functionalization of *SiATLb1/6/10* (SD3), *SiBAT1/SiBAT8* (SD8) and *SiCAT3/10* (SD9), and non-functionalization of *SiATLa1/5* (SD1), *SiLHT1/2* (SD6) and *SiCAT4/11* (SD10) (Fig. 6b).

**Fig. 7:**
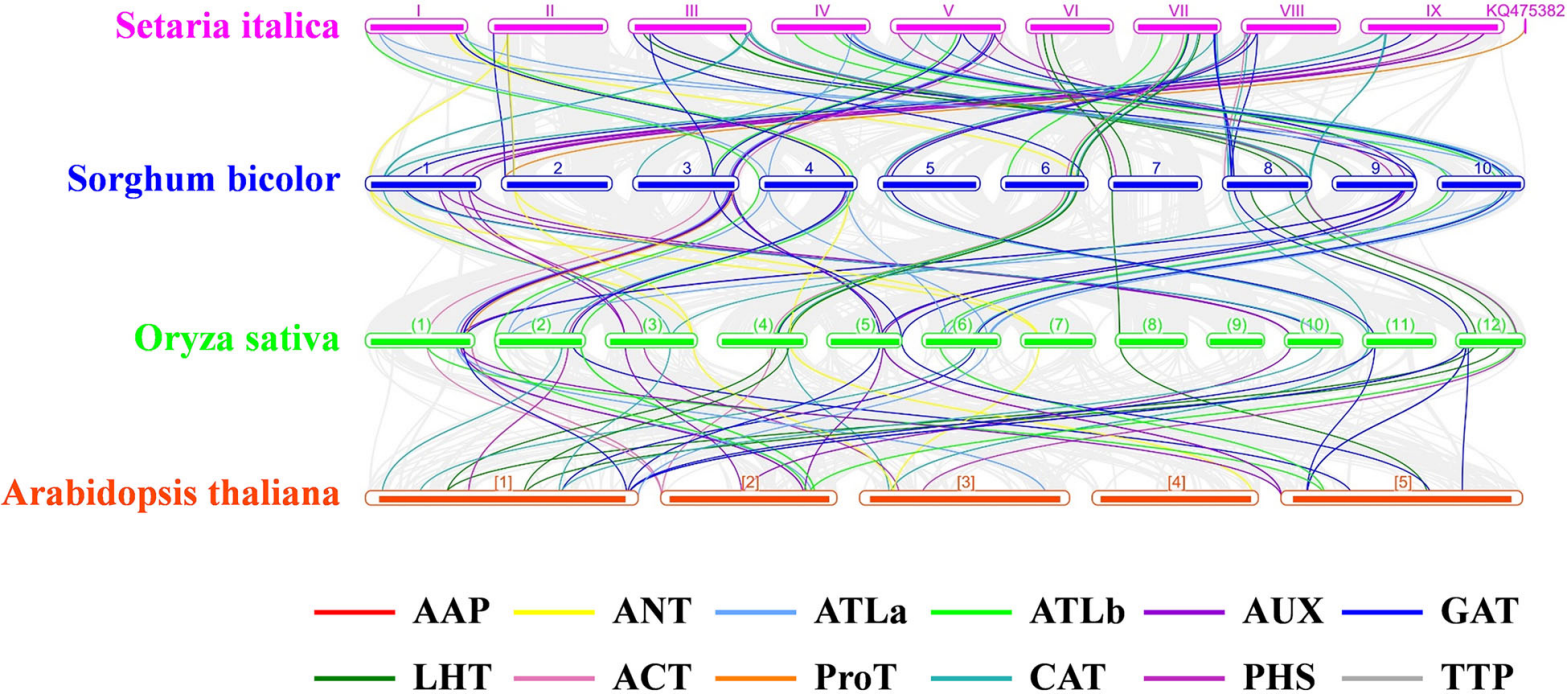
Collinearity of the orthologous *AAT* genes in foxtail millet, sorghum, rice and *Arabidopsis*. The genome of each species is shown in one row, and the *AAT* genes of different subfamily are shown with different colored lines. The collinear relationship of all orthologous genes in different species was shown with the gray lines

**Fig. 8:**
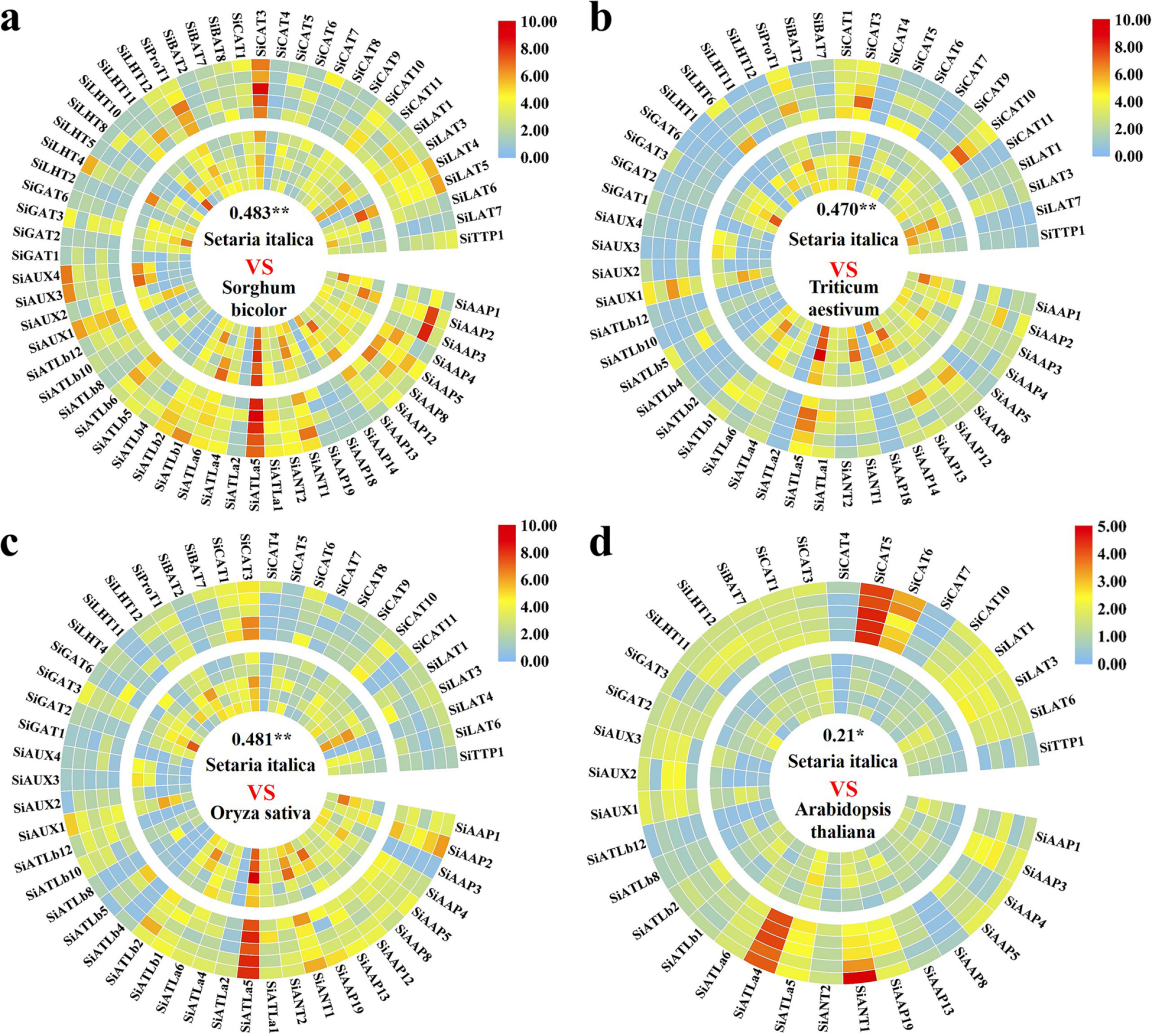
Comparison on the expressions of orthologous *AAT* genes in five main tissues between foxtail millet with other 4 species. The outside and the inside rings of (**a**) to (**d**) represent foxtail millet and another species, respectively. From the outside to the inside of each ring are the expression levels of *AAT* genes in leaf, root, stem, grain and spike/inflorescence, respectively. The numbers in the circles represent the correlation coefficients between the expressions of orthologous *AAT* genes in the two species. * and ** represent correlation significant at the *P* < 0.05 and *P* < 0.01 probability level, respectively

There were 62, 51, 52 and 35 orthologous *AAT* genes found in sorghum, rice, wheat and *Arabidopsis*, respectively, and their corresponding relationships with foxtail millet were investigated (Fig. 7; Additional file 6: Table S2**).** The wheat genome was much larger than other species, so it was not displayed in Fig. 7. The collinear relationship between different species was clearly distinguishable and showed good collinearity between chromosome 9 of foxtail millet and chromosome 1 of sorghum, chromosomes 3, 4, 5 of foxtail millet and chromosomes 8, 9, 10 of sorghum, respectively, etc. Moreover, the collinearity of the orthologous *AAT* genes among different species was consistent with that of the whole genomes. The number of orthologous subfamilies *LHTs* and *LATs* in millet and sorghum were significantly more than those in wheat and rice, which suggested that their expansion in millet and sorghum completed later than that in sorghum and wheat (Fig. 8; Additional file 6: Table S2). Compared with *Arabidopsis*, the AAP subfamily in grass species was significantly expanded. For instance, the orthologous of *SiAAP2*, *SiAAP12* and *SiAAP14* only could be found in grass species, which suggested that they might be produced after the differentiation of monocotyledon and dicotyledon.

The expression characteristics of those orthologous *AAT* genes in root, stem, leaf, inflorescence/spike and grain of foxtail millet, sorghum, wheat, rice and *Arabidopsis* were compared using their intergrated transcriptome datasets. Owing to the heterohexaploid nature of wheat with almost three homologous copies for each gene, the average expression values of these copies were used. The expression patterns of *SiAATs* in foxtail millet and their orthologous in other species were generally conservative, especially in the grass species (Fig. 8). The correlation coefficients on the expression patterns of *AAT* genes in gramineous species were 0.483, 0.470, and 0.481 between foxtail millet with sorghum, wheat and rice, respectively, while it was only 0.21 between foxtail millet and *Arabidopsis*. Several high-expressed *AAT* genes in foxtail millet were also highly conserved in other species, such as *SiCAT3* and *SiATLa5*. In addition to the relatively conservative expression patterns of some important orthologous *AAT* genes among different species, the expression patterns of some genes were differentiated, such as *SiATLa6* and *SiATLb6* (Fig. 8). Compared with the orthologous genes in *Arabidopsis*, *SiAATs* showed greater variation in expression patterns, and several genes changed their tissue specificities, such as *SiATLb1* and *SiAUX3*.

### Spatiotemporal expression patterns of *SiAATs*

**Fig. 9:**
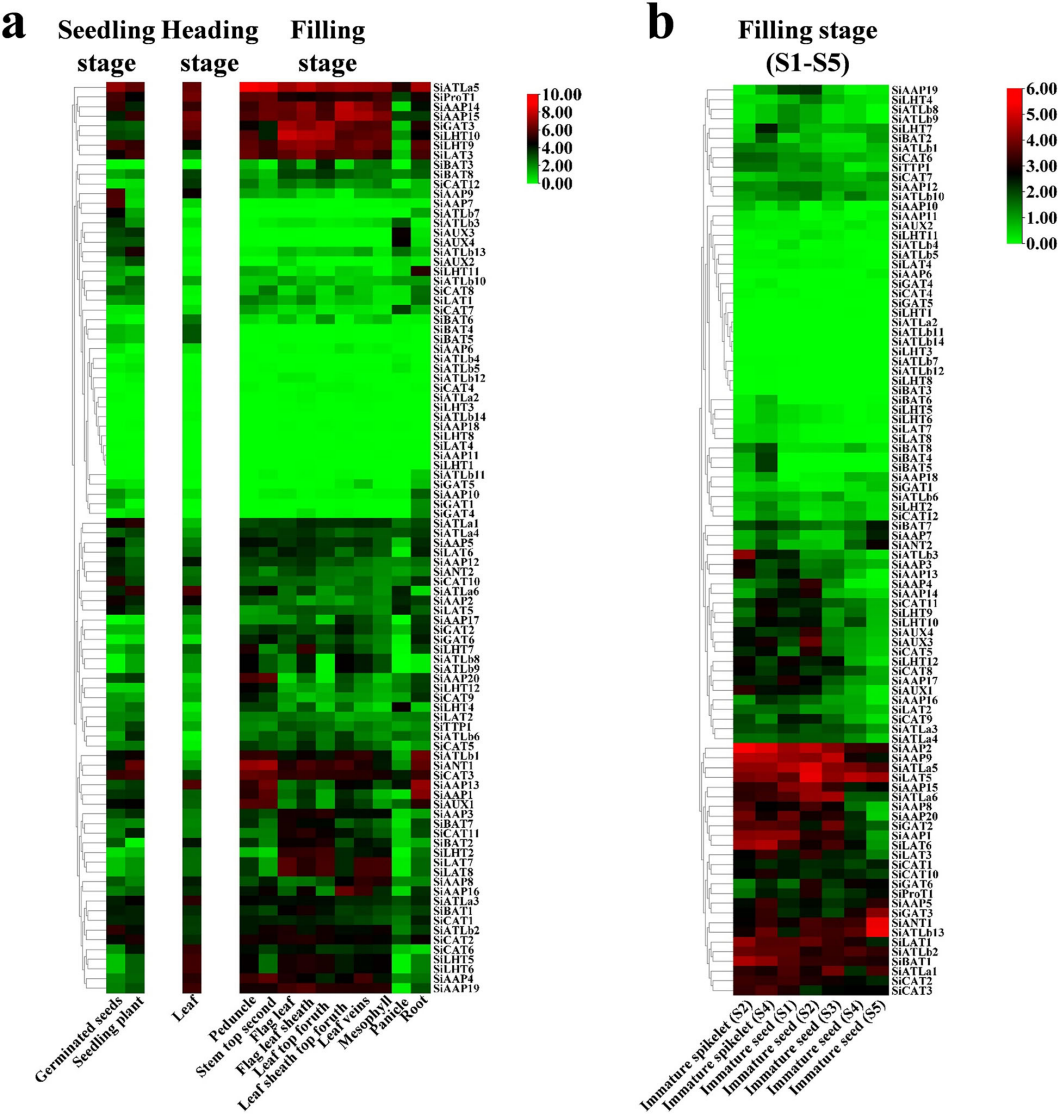
Expression patterns of *SiAATs* in multiple tissues and developing grain of foxtail millet. (**a**) The expression patterns of *SiAAT* genes in 13 tissues at three growth stages, which were reproduced based on the transcriptome data; (**b**) The expression pattern of *SiAATs* in developing grain, which were obtained from RNA-seq. Tissue name is listed at the bottom of each column, while the color scale is shown at the right. The log2 (FPKM+ 1) values are used to display the expression levels

The expression patterns of *SiAATs* were analyzed using the RNA-seq data of different tissues at mutiple growth stages collected from the public online database. The heat map was displayed with the normalized log_2_ (FPKM+ 1) values (Fig. 9a). According to the expression patterns, the *SiAATs* were clustered into three groups. The 8 *SiAAT* genes in the first group included 7 AAAP family genes and one APC family gene with relatively high expression level, and were stably expressed in almost all tissues at different developmental stages. The 37 *SiAAT* genes in the second group were expressed in low abundance in most tissues, but expressed explicitly in some tissues, such as *SiAAP9* and *SiAAP7* were highly expressed in germinating seeds, while *SiATLb3*, *SiAUX3* and *SiAUX4* were relatively high expressed in panicle. The 49 *SiAAT* genes in the third group showed diverse spatiotemporal expression characteristics, such as *SiATLa6* and *SiATLa3* were highly expressed in the leaves at the seedling stage, but lower in the leaves at filling stage; *SiAAP20* was specifically high-expressed in the stem, while *SiAAP3*, *SiBAT7*, *SiCAT11*, *SiBAT2*, *SiLAT7* and *SiLAT8* were expressed abundantly in leaf tissues including leaf sheath and mesophyll. In addition, some genes were highly expressed in two or more tissues, such as *SiAAP1*, *SiAAP13* and *SiAUX1* in stem and root; *SiANT1*, *SiCAT3* and *SiATLb1* were expressed abundantly in organs involved in the entire source-sink circulation including root, stem, leaf and grain. The expressions of the 20 selected *SiAATs* by qRT-PCR analysis were not completely consistent with that from the transcriptome data, but their expression characteristics and trends were similar, which verified the reliability of the transcriptome data (Fig. 9a; Additional file 4: Fig. S4).

**Fig. 10:**
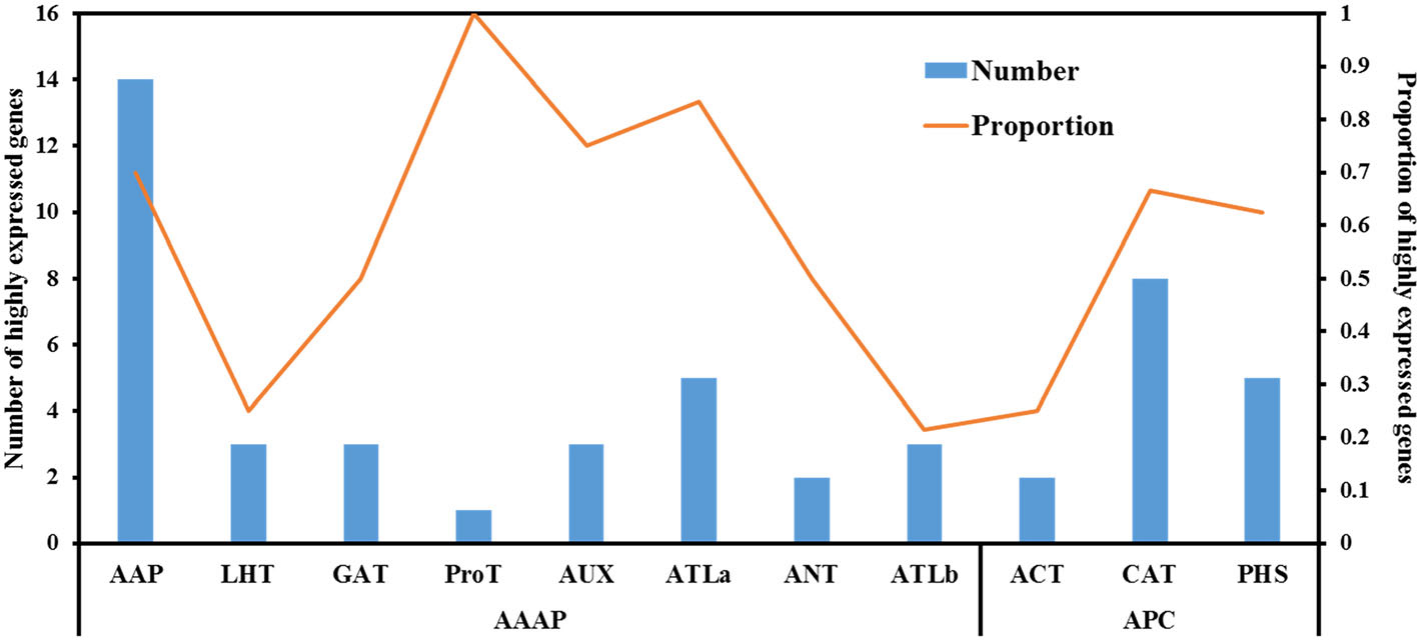
The numbers and proportions of the *SiAAT* genes in different subfamilies specifically expressed in grain development. The bars represent the number of genes in different subfamilies, and the broken line represents the rate of grain-specific expressed genes to the total number of genes in the subfamily

The transcriptome analysis of spike RNA-seq data showed that about half of *SiAATs* were expressed lowly during seed development, while the remaining ones were expressed at relatively high levels at different stages (Fig. 9b). *SiAAP2*, *SiAAP9*, *SiATLa5*, *SiLAT5*, *SiATLb2* and *SiBAT1 etc,* were highly expressed in spikelets and grains throughout the grain development process (from S1 to S5). *SiATLa6*, *SiAAP8*, *SiAAP20*, *SiAAP1*, *SiLAT6*, *SiCAT2* and *SiCAT3, etc* were highly-expressed at the early stage of grain development. *SiGAT6* and *SiProT1, etc* were highly expressed at specific stages. In addition, the numbers of the highly expressed *SiAATs* during grain development in different subfamilies were counted, as 14 *SiAAPs*, 8 *SiCATs*, 5 *SiLATs*, 5 S*iATLas* and 3 *SiAUXs*, which accounted for 70% of total *SiAAPs* (20), 67% of *SiCATs* (12), 63% of *SiBATs* (PHS, 8), 83% of *SiATLas* (6) and 75% of *SiAUXs* (4), respectively (Fig. 10).

**Fig. 11:**
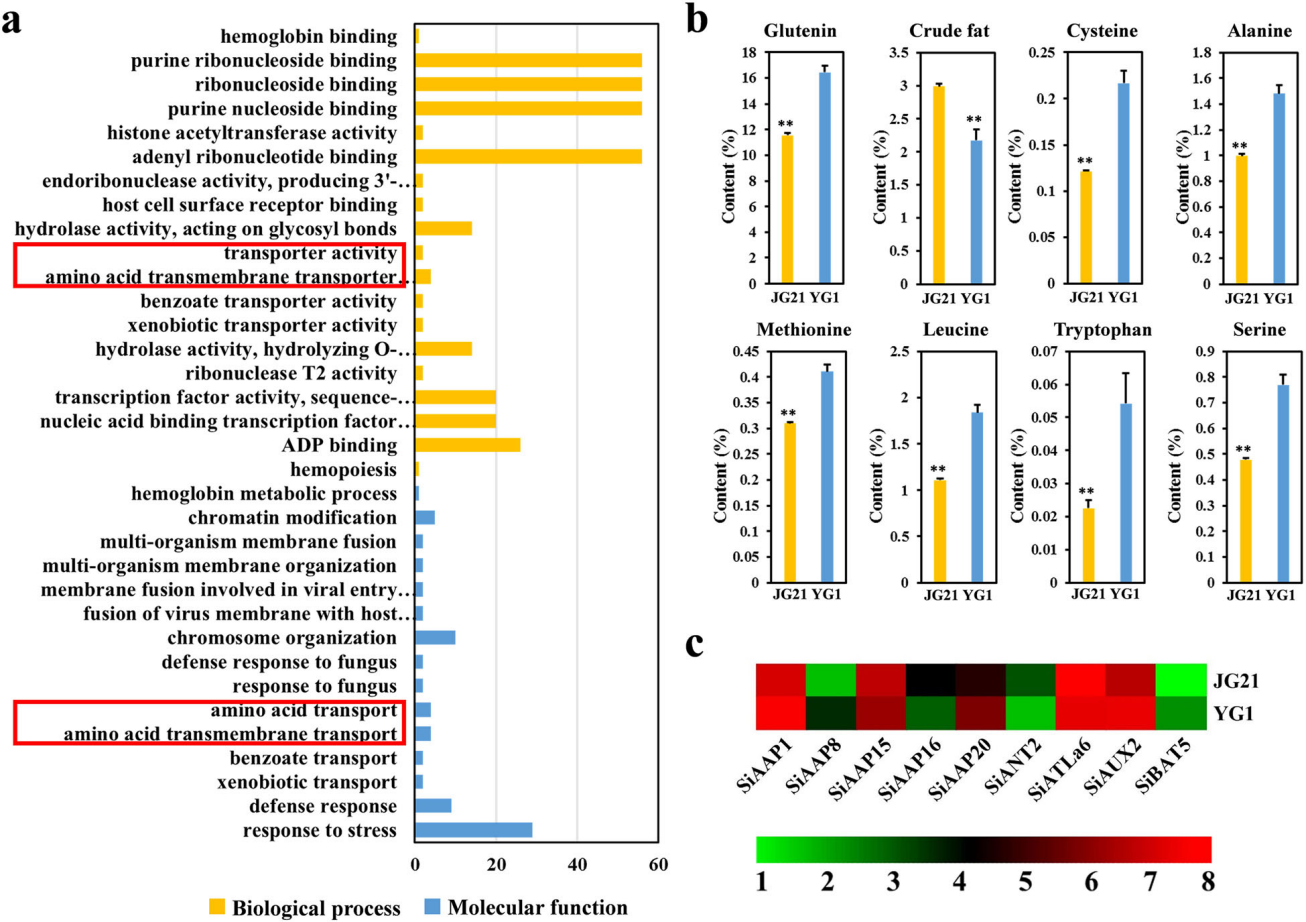
The comparative analysis on the transcriptome in grain development of two millet genotypes. (**a**) The GO enrichment of differently expressed genes (DEGs), the four terms related to amino acid transport were indicated with red boxes. (**b**) The differences in grain quality traits between “JG21” and “YG1”. ** represents the differences significant at *P* < 0.01. (**c**) The expression of 9 *SiAATs* in the grains of “JG21” and “YG1”

There were significant differences in multiple grain quality traits between the two foxtail millet genotypes “JG21” and “YG1”. Except for crude fat content, the basic amino acid contents of glutenin, cysteine, alanine, methionine, leucine, tryptophan and serine in “JG21” grain was significantly lower than “YG1” (Fig. 11b). The developing grains (including glumes) of “JG21” and “YG1” at filling stage in 2019 were separated for RNA extraction and RNA-seq. Finally, 348 differentially expressed genes (DEGs) in the developing grains of these two genotypes were identified, of which 164 and 184 DEGs were down-regulated and up-regulated in “JG21”, respectively. GO analysis showed that DEGs were mainly enriched in multiple terms such as responses to fungus or viruses, ribonuclease activity, stress response, and transmembrane transport of various substances (Fig. 11a). Interestingly, 9 *AAT* genes were enriched into four terms related to amino acid transport, including 5 *SiAAPs*, 1 *SiANT*, 1 *SiATLb*, 1 *SiAUX* and 1 *SiBAT* (Fig. 11c). The differential expression of these *SiAATs* suggested that these genes might directly affect the formation of grain quality.

### Response of *SiAATs* to abiotic stresses

**Fig. 12:**
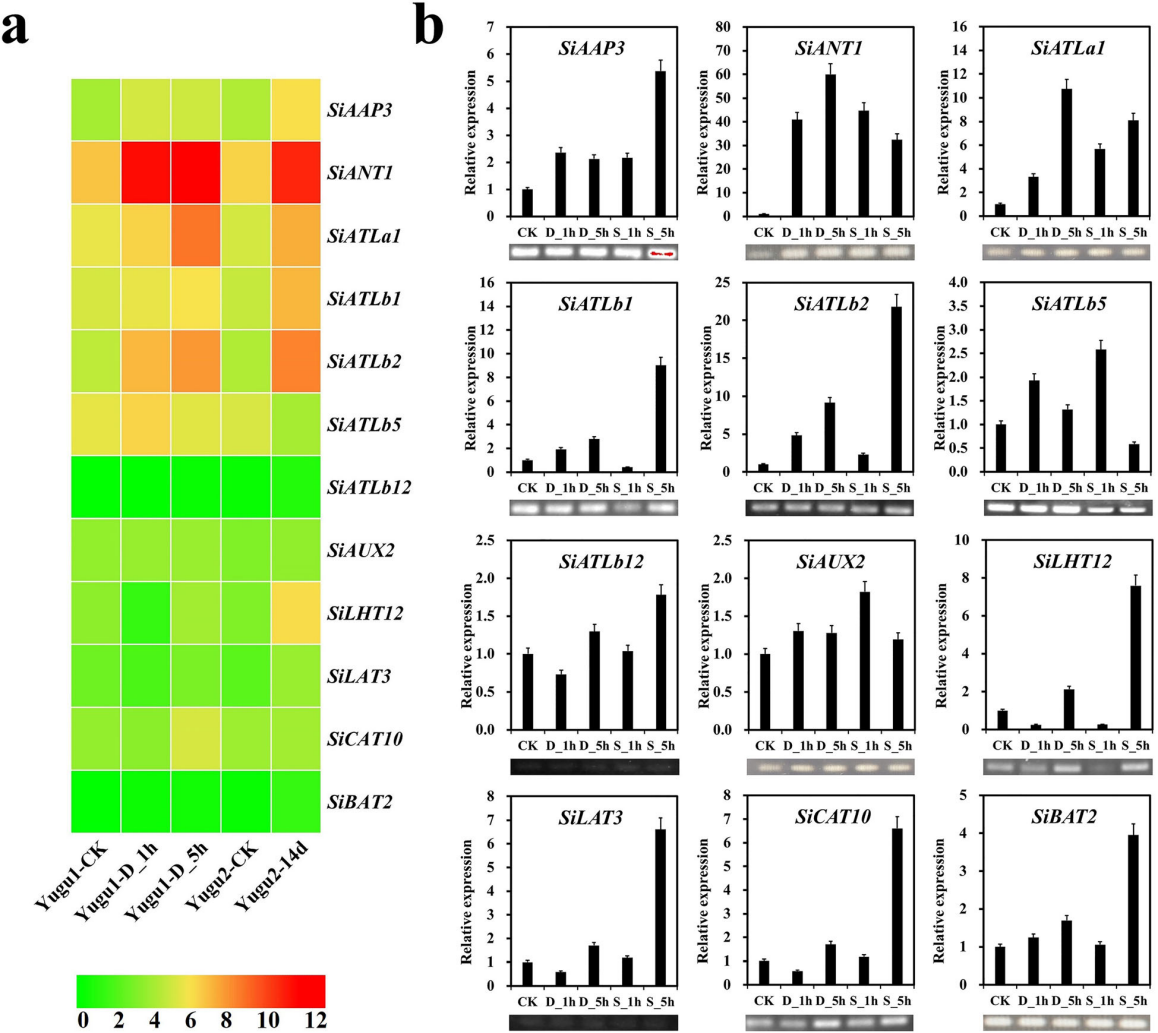
The expression patterns of 12 selected *SiAAT* genes under drought and salt stresses. (**a**) The RNA-seq results of 12 *SiAATs* under drought and salt stresses, which are reanalyzed with the public transcriptome data SRA062640 and PRJNA545871; (**b**) The qRT-PCR results of these 12 *SiAATs*. The seedlings 15 days after sowing were exposed to 20% polyethylene glycol (PEG 6000) solution and NaCl solution (200 mM) for 1 h and 5 h, respectively. Bars represent the mean values of three replicates ± standard deviation (SD). The *SiAct-7* gene is used as an internal reference. The electropherograms of all PCR amplified products are also displayed in the corresponding position

The qRT-PCR results of JG21 and the transcriptome data of the foxtail millet cultivars of Yugu1 and Yugu2 showed a relatively consistent trend of a similar response of *SiAAT* genes to abiotic stress (Fig. 12a). The qRT-PCR analysis of 12 *SiAATs* in foxtail millet seedlings (15 days after sowing) to simulate drought (20% PEG 6000, 1 h and 5 h) and salt (200 mM NaCl, 1 h and 5 h) stress revealed that various subfamilies of *SiAATs* responded differently to abiotic stresses (Fig. 12b). Seven *SiAAT* genes were up-regulated more than 5 times after drought or salt stress, of which *SiANT1* was up-regulated by about 60 times at 5 h after drought stress. *SiANT1*, *SiCAT10* and *SiATLa1* were mainly induced by drought stress, and their expression all reached the highest levels at 5 h after drought treatment. Except for *SiAUX2* and *SiATLb5*, the other 7 *SiAAT* genes reached their highest expression levels at the late stage of salt stress (5 h). *SiLHT12*, *SiATLb1* and *SiLAT3* were specifically and strongly induced by long-time salt stress, while *SiANT1*, *SiATLa1* and *SiATLb5* had similar expression patterns under both drought and salt stress.

The response of different *SiAATs* to abiotic stress also showed temporal specificity. *SiAAP3* was up-regulated under 1 h drought stress, while it was up-regulated at 5 h under salt stress. *SiLHT12* was down-regulated under 1 h drought and salt stresses, but then was strongly up-regulated at 5 h (Fig. 12b). *SiANT1* was continually up-regulated with an increase of drought stress, and was down-regulated under 5 h salt stress. *SiATLb5* and *SiATLa1* responded at the early and late stages of drought and salt stress, respectively. Furthermore, some *SiAATs* showed a sustainable response to abiotic stress; for example, both *SiATLb2* and *SiATLa1* were continually up-regulated under both salt and drought stress. These genes might enhance the adaptability of foxtail millet to abiotic stresses through their active responsiveness.

## Discussion

### A large number of tandem and segmental duplication events were the direct cause of *AAT* gene family expansion in foxtail millet

**Fig. 13:**
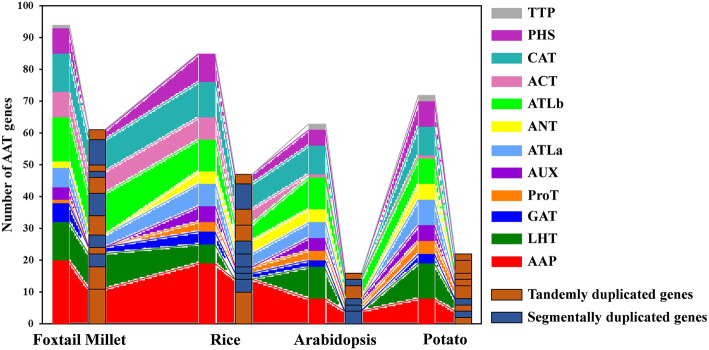
Distribution of the numbers of duplicated genes in different AAT subfamilies in foxtail millet, rice, *Arabidopsis* and potato. The number of all *AAT* genes and the number of *AAT* genes involved in the duplication event were in the column on the left and right of each species, respectively. The paralogous *AAT* genes generated by different duplication events and the *AAT* genes of different subfamily were marked with different colors. The same color connects the paralogous genes corresponding to the same subfamily

Amino acid transporters (AATs) play indispensable roles in plant growth by transporting and distributing different types of amino acids, and therefore are considered as important targets for crop improvement [15]. Here, a total of 94 *AAT* genes were characterized in foxtail millet, which were divided into 12 subfamilies and was consistent with the previous reports in other plants (Table 1). Different subfamilies of *AATs* in monocotyledons and dicotyledons were corresponding, thus confirming that they were formed before the differentiation in monocotyledons and dicotyledons (Fig. 2). The number of *SiAATs* in foxtail millet was less than those in hexaploid wheat (294) and maize (107) [13], but more than that in sorghum (77), rice (85) [12], and *Brachypodium* [13], and much higher than that in *Arabidopsis* (63) [1] and potato (72) [10]. Earlier studies indicated that the plant *AAT* gene family originated from the prokaryotic genome through horizontal gene transfer and subsequent duplication events [49]. There were 47, 16, 22 *AAT* genes associated with duplication events in rice, *Arabidopsis* and potato, accounting for 55.29% (47/85) [12], 25.40% (16/63) [10] and 30.55% (22/72) [10], respectively. As observed in this study, 58 of the 94 (62%) *SiAATs* were duplicated in foxtail millet, including 36 and 25 tandemly and segmentally duplicated genes, respectively. All these further verified the influence of gene duplication of the AAT family expansion in monocots. Furthermore, obvious imbalances in the expansion of different *AAT* subfamilies in monocots and eudicots were found, as AAP, ATLa/b, ACT and CAT subfamilies expanded greatly in monocots, while more *ProT* genes in eudicots (Table 2; Fig. 13). These increased AATs may play important roles in specific functions, and promoted further differentiation of monocots and eudicots.

### The multi-levels variations of paralogous *AAT* genes generated from gene duplication promoted the functional diversity of AAT family in foxtail millet

Gene duplication and subsequent functional differentiation of new genes promoted the expansion and new-functionalization of important gene families, thereby enabling plants to better adapt to the environment [46]. Variations in the sequence, structure and expressions of genes were the three main sources of functional differentiation [13]. Base mutation was the most direct evidence of sequence variation. In general, the Ka/Ks values of all duplicate groups except for TD12 were far less than 1, indicating that most of the paralogous *AAT* genes undergone purifications in sequence variation to ensure the stability of key biological functions (Fig. 5). The Ka/Ks values of different AAT subfamilies showed different median and dispersion, such as AAP, PHS and CAT subfamilies had higher dispersion, which indicated that these subfamilies may have the opportunity to produce new features. However, the Ka/Ks values of ACT subfamily, both tandemly and segmentally duplicated gene pairs, showed smaller dispersion, suggesting that their functions might be more conservative. In addition, tandemly duplicated gene pairs showed higher Ka/Ks values than segmentally duplicated gene pairs, suggesting they were under stronger selection pressure, which confirmed that tandem duplication played more crucial roles in functional diversity of foxtail millet *AAT* genes. Similar results were also found in the study of wheat *AAT* family genes [13]. These results confirmed that the sequence variations of paralogous genes in different AAT subfamilies of foxtail millet increased the diversity of *AAT* genes.

Previous studies have shown that variations in gene structure are also important markers of gene family evolution [48, 49]. In this study, 56% (14/25) of the paralogous gene groups had significant changes in the number of introns, resulted in 9 and 5 groups of tandem and segmental duplications, respectively, accounting for 69 and 42% of the total (Table 2; Additional file 3: Fig. S3). The variations in the number of TM regions were found in 77% (10/13) and 42% (5/12) of the tandemly and segmentally duplicated gene groups, respectively, which were caused by the deletion of conserved domains, such as SiAAP5/6/7 (TD1), SiAAP8/9 (TD2) (Fig. 1b; Fig. 4). There were significant variations in gene structure and the number of TM regions which was observed in 56% (14/25) and 60% (15/25) of the two duplicated gene groups, confirming that the structural variation of paralogous genes was one of the important sources of the functional categorization of the *AAT* family genes in foxtail millet.

Changes in expressions of paralogous genes are another key sources of functional categorization of important gene family [49]. Sub-functionalization and new-functionalization of new duplicated genes directly lead to significant differences in expression level and spatiotemporal expression patterns, compared to their ancestor genes [48]. As observed in rice [12], soybean [11] and potato [10], the changes of the three expression types of paralogous genes were also observed in this study. For instance, *SiAAP8/9* (TD2) and *SiAAP19/20* (TD4) were specifically expressed in different tissues, (Fig. 6), confirming that the expression changes of paralogous genes in AAT family were common in higher plants. Interestingly, the paralogous genes with new functions were only found in tandemly duplicated gene groups, suggesting that tandem duplication has a greater contribution to the evolution of the *AAT* gene family in foxtail millet.

### The changes of orthologous *AAT* genes in foxtail millet play important roles in differentiation of new functions

Along with the separation of species and the influence of natural selection, though the orthologous genes of different species are largely conserved, the variations in their regulatory elements, epigenetic markers, *etc* will cause changes in the pattern of expression of orthologous genes in various species, resulting in their functional differentiation [50–54]. For some C_4_ genes in C_4_ species, such as *PEPC*, *MDH* and *PPDK*, their C_4_ functions were directly obtained by the differences in the subcellular localization and expression level of orthologous genes [55]. The completion of genome sequencing allowed us to quickly identify the orthologous genes of different plants at genome level (Fig. 7). There were 62, 51, 52, 35 orthologous of the *SiAATs* identified in sorghum, rice, wheat and *Arabidopsis*, which were consistent with the genetic distance and differentiation time of these species [56]. The AAP subfamily of grass species expanded significantly after the differentiation of monocots and eudicots, which was consistent with previous studies [12, 13]. Within the grass family, the LHT and LAT subfamilies expanded significantly in foxtail millet and sorghum, which may have improved their adaptability to the environment. Most *SiAATs* and their orthologous in other species had similar expression patterns, especially for those of grass family with high correlation coefficients ranged from 0.470 to 0.483 (Fig. 8). Compared with their orthologous in *Arabidopsis*, the tissue expression characteristics of some *SiAATs* had changed, which may be related to the long genetic distance and complete differentiation between foxtail millet and *Arabidopsis*.

### Many important *AAT* genes were expressed conservatively in multiple species to retain essential biological functions

Spatiotemporal expression analysis of important gene family members will help to predict their putative functions [57]. Some *SiAATs*, such as *SiProT1* and *SiATLa5*, were abundantly expressed during the whole period of growth, which were essential for the growth and development of foxtail millet (Fig. 9). Some *SiAATs* were abundantly expressed in specific organs at certain developmental stages, such as *SiAAP9*, *SiAAP7* and *SiAAP2* in germinating seeds, *SiAAP1* and *SiAUX1* in stems and roots, and *SiLAT7* and *SiLAT8* in leaf tissues, while, *SiAAT6* was highly expressed only in leaves at seedling and heading stages, but low at filling stage. The different spatiotemporal expression patterns of these genes suggested that they might have specific functions.

The functions of some *AAT* genes have been extensively studied in the model species, such as *AtAAP1* for seed germination and amino acid intake in root [9, 14, 58]. It was found that *SiAAP7* and *SiAAP2*, *OsAAP1* [26] possessed similar functions; *AtAAP3* in the uptake of amino acids in xylem, and *SiAAP4* highly expressed in stems and veins [21]; *StAAP1* was expressed in multiple organs of source-sink transport in potato [10, 24]; *AtLHT1* located in root epidermis and mesophyll cells in *Arabidopsis*, which was primarily responsible for the amino acid uptake in soil [28, 29]; *AtAUX1*, *AtLAX3* and *OsAUX1* related to the gravitropism of root and lateral root formation [12, 32, 33]. As similar expression patterns were also observed for orthologous genes, which maintain the important process of plant growth and development. Therefore, we preliminary predicted their functions in foxtail millet based on these previous studies, the phylogenetic relationships and the spatiotemporal expression characteristics of *SiAATs* in foxtail millet (Table 3).

**Table 3:** Functional prediction of *AAT* genes in foxtail millet based on homology relationship, expression pattern and previous studies

**Subfamilies**	**AATs in foxtail millet**	**expression organization**^**a**^	**Possible biological processes**	**Previous studies**
AAP	SiAAP7;	GS;	The intake of amino acid in endosperm and embryo development;	AtAAP8,1 [23, 58];
	SiAAP1,5,13;	R,ST;	Amino acid absorption in root and transport in stems;	AtAAP7,1 [19];
	SiAAP2,10,9;	GS,SP,G	Play a role in early embryo development and formation of floral organs and grain quality;	AtAAP8,1,VfAAP1,OsAAP6 [4, 6, 7];
	SiAAP3,8,12,14	LS,LV, G	Participate in the transport and distribution of amino acids in leaf and grain formation;	
	SiAAP4,15,19,20	ST,LS,LV	Participate in the transfer and distribution of amino acids from stems to leaf	HvAAP3,AtAAP7 [25];AtAAP2,3,6,OsAAP8,15;HvAAP2,6 [8, 12, 20, 21]
GAT	SiGAT3;	LS,LV,L,SP	Maintain cell ion balance under normal conditions;	AtGAT1 [44]
	SiGAT6	R,ST,LS,SP	Amino acid absorption in root and long-distance transportation of GABA	
ProT	SiProT1	ST,L,GS	Participate in the long-distance transport of proline and regulate the distribution of proline in leaf	AtProT1,AtProT3 [5, 38]
LHT	SiLHT11	R;	Amino acid uptake in root;	AtLHT1,9 [28, 59]
	SiLHT2,5,10	L;	Transport and distribution of amino acids in leaf;	
	SiLHT9	LS,ST,R	Long-distance transportation of amino acids from root to leaf	
AUX	SiAUX1;	ST,R;	Participate in the geotropism of root and the transport of auxin in the stem;	AtAUX1 [32–34];
	SiAUX2,3,4;	SP; G	Participate in spikelet development and grain formation	OsAUX1,StAUX [10, 12]
ANT	SiANT1;	ST,R,LS,LV;	Participate in long-distance transportation of amino acids;	AtANT1 [31]
	SiANT2	SP,G,GS	Participate in seed germination, spike differentiation and grain formation	
ATLa	SiATLa1;	GS,SE;	Participate in seed germination;	NA
	SiATLa3,4,5;	ST,LS,LV,R,SP;	Participate in long-distance transportation of amino acids;	
	SiATLa6	L,SP	Regulate the distribution of amino acids in leaf and Participate in spike differentiation	
ATLb	SiATLb3,6,10,13;	GS,SE,SP,G;	Participate in seed germination, spike differentiation and grain formation;	NA
	SiATLb1;	R,ST,LV;	Long-distance transportation of amino acids from root to leaf;	
	SiATLb7;	GS;	Participate in seed germination;	
	SiATLb8;	ST,LS,LV	Long-distance transportation of amino acids from stem to leaf;	
CAT	SiCAT10	GS	Participate in seed germination;	NA
	SiCAT3, 5, 6, 8, 9	GS, ST, R, L	Participate in long-distance transportation of amino acids;	
	SiCAT7	SP	Specific influence on spike differentiation;	
	SiCAT11, 12	SP, L	Participate in spikelet formation and amino acid transport and distribution in leaves	
ACT	SiBAT1, 2, 7	ST, L, SP	Participate in spikelet formation and long-distance transportation of amino acids	NA
PHS	SiLAT1, 2, 5	GS, SP, G;	Participate in seed germination, spike differentiation and grain formation;	NA
	SiLAT3	GS, LS, LV, ST, R	Participate in spikelet formation and long-distance transportation of amino acids	
TTP	SiTTP1	SE, SP	Affect seedling growth and spikelet differentiation	NA

^a^*GS* Germinated seed, *R* Root, *ST* Stem, *SP* Spikelet, *G* Grain, *SE* Seedling, *L* Leaf, *LV* Leaf Vein, *LS* Leaf Sheath

### Multiple *AAT* genes may contribute directly to grain quality traits in foxtail millet

The *AAP* genes are closed related to grain development and quality formation in many species. *AtAAP1* in *Arabidopsis* and *OsAAP6 in* rice are directly related to grain protein content [7, 14], multiple *AAT* genes were located in or near the QTL regions associated with quality traits in wheat by genome-wide association analysis (GWAS) [27]. The RNA-seq analysis of the foxtail millet grains at development stages revealed that 70% *AAPs* (14/20), 67% *CATs* (8/12), 63% *BATs* (5/8), 83% *ATLa* (5/6) and 75% *AUXs* (3/4) were highly expressed during grain development, including *SiAAP9* and *SiAAP8,* the orthologous genes of *OsAAP6*, suggested that they played a major role in the development and quality formation of foxtail millet grain (Fig. 10). The DEGs between the two cultivars with extremely significant differences in the content of glutenin, crude fat, four non-polar amino acids (Alanine, Methionine, Leucine and Tryptophan) and two neutral amino acids (Cysteine and Serine) were enriched into four GO terms related to amino acid transport (Fig. 11a). Nine *SiAATs*, including *SiAAP8*, belonging to AAP, ANT, ATLa, AUX and PHS subfamilies, were expressed differentially in the developing grains of the two genotypes, which suggested that they might contribute to the grain quality traits (Fig. 11c). In *Arabidopsis*, *AtAAP1* and *AtAAP2* directly affected the grain protein content, and *AtAAP2* also significantly affected the fat content in the grain [20]. The differential expression of their homologs *SiAAP1* and *SiAAP8* in the two foxtail millet grains may lead to their difference in grain protein and crude fat content. *Arabidopsis AtAAP6* mutant showed a decrease in the content of lysine, phenylalanine, leucine and aspartic acid [8]. Therefore, it is speculated that the difference in leucine may be caused by the differential expression of *SiAAP20*, a homologous gene of AtAAP6 in foxtail millet. In addition, neutral amino acid transporters (ANTs) could specifically transport aromatic and neutral amino acids. The content of such amino acids including cysteine, tryptophan and serine may be affected by the expression level of *SiANT2*. However, the functions of those differentially expressed *SiAAT* genes on the contents of different amino acids and protein content in foxtail millet grains should be verified in detail in further experiments. All these suggested that it might be feasible to improve the quality traits by manipulating the expressions of those important *SiAAT* genes in foxtail millet, and our results provided a base for further analysis of their function in quality formation in foxtail millet.

### *SiAATs* enhanced the adaptability of foxtail millet to abiotic stresses

Drought and salt seriously affect crop yield and grain quality, and are the most important abiotic stresses in agricultural production. In *Arabidopsis* and other species, it has been confirmed that *AAT* genes in higher plants regulate the balance of osmotic potential to resist the impact of stress on plant growth, mainly through the transport of a variety of stress-response compounds and compatible solutes, such as betaine, GABA, and proline [40–44]. *AtAAP4* and *AtAAP6* were down-regulated under salt stress [42], and *OsAAP4*, *OsAAP8* and *OsBAT4* were down-regulated under both salt and drought stress [12]. *AtProT2* [42], *McAAT1* [60] and *HvProT* [61] were greatly triggered by salt stress. In this study, active responsiveness to simulated drought and salt stresses were observed in 12 *SiAAT* genes investigated, and various response patterns to different stresses were found in different subfamily members (Fig. 12). It should be noted that *SiAATs* located on vacuole membranes, such as *SiANT1* and *SiALTb2,* responded stronger to these abiotic stress, which may relate to their roles in rapid adjustment of cell osmotic potential to maintain cell water balance. These genes could be used as key candidate genes to improve resistance and tolerance under abiotic stresses.

## Conclusions

In this study, 94 *AAT* genes in foxtail millet were systematically identified and characterize, which were divided into 12 subfamilies. The paralogous genes generated by tandem duplication and segmental duplication promoted *AAT* gene family expansion. The variations in sequence, structure and the pattern of expression of tandemly duplicated paralogous *AAT* genes had significant impacts on their functional differentiation. The functional differentiation of *SiAATs* was the result of the unequal expansion and differentiation of paralogous genes in different subfamilies. The transcriptome analysis revealed the contributions of some *AAT* genes in the formation of millet quality. Multiple *SiAAT* genes actively responded to drought and salt stresses as revealed by qRT-PCR. The possible functions of the *SiAAT* family members were predicted. These findings might provide valuable information for further functional analysis of *AAT* genes and their applications in the improvement of foxtail millet.

## Methods

### Genome-wide scan and identification of *AAT* genes in foxtail millet

The latest foxtail millet genomic and protein sequences (*Setaria italica* v2.0, release-41) were obtained from the ensemble plants database (http://plants.ensembl.org/info/website/ftp/index.html). First, the AAT proteins identified in *Arabidopsis*, rice and wheat were aligned with the millet protein sequences by BLASTP program with an e-value of 1e^− 5^ and an identity of 50% as the threshold. Second, the conserved domains of putative AAT proteins were checked using the Hidden Markov Model (HMM) profiles of the AAT domain (PF01490 and PF00324) from the Pfam database (http://pfam.xfam.org/), by a HMMER tool with a ‘hmmsearch’ command [62]. Third, the existence and integrity of the conserved domains in candidate proteins were further analyzed through conserved domain database (CDD) (https://www.ncbi.nlm.nih.gov/cdd) and InterPro database (http://www.ebi.ac.uk/interpro/scan.html) with default parameters. Finally, the *AAT* gene family members in foxtail millet were identified after removing those containing incomplete conservative domains and redundant sequences.

Based on the chromosome position and phylogenetic relationship, these putative *AAT* genes in foxtail millet were systematically named, and their detailed information was counted, including cDNA length, coding sequence length, gene structure and protein sequence feature. Their gene structures and biochemical parameters were determined by GSDS (http://gsds.gao-lab.org/) with the genome annotation GFF3 file and Computer pI/Mw tool (https://web.expasy.org/compute_pi/), respectively [63]. As complete membrane proteins, their transmembrane (TM) regions and subcellular localization were predicted by TMHMM server 2.0 (http://www.cbs.dtu.dk/services/TMHMM/) and LocTree 3 (https://www.rostlab.org/services/loctree3/) using the default parameters, respectively [64].

### Chromosomal mapping, duplication and selective pressure analysis

The locations of *SiAAT* genes on the chromosomes were obtained from the ensemble plants database. The gene duplication was determined by MCScanX, and manual screening was performed according to Wang et al. [65, 66]. The distributions of *AAT* genes and their duplicated paralogous on chromosomes were ploted with TBtools software [67]. The ratios (Ka/Ks) of non-synonymous (Ka) and synonymous (Ks) of these paralogous *AAT* genes were analyzed using KaKs_Calculator 2.0 and displayed by an R package ggplot2 followed the standard processes as detailed in the software manuals [68].

### Phylogenetic and conserved motifs analysis

Multi-sequence alignment of the AAT protein sequences identified in *Arabidopsis*, potato, rice and foxtail millet was conducted with ClustalW, and phylogenetic tree was created using the maximum likelihood (ML) method, JTT model and a bootstrap of 1000 using MEGA 6.06 [69]. The phylogenetic tree was displayed with iTOL v3 (http://itol.embl.de/#).

The conserved motifs were determined using Motif-based sequence analysis (MEME) program (http://meme-suite.org) with the parameters of motif width of 6–200, the maximum motif number of 20 and any number of repeating motif sites [70]. TM regions and the possible protein secondary structures obtained from Protein Data Bank (PDB) database (https://www.rcsb.org/) were annotated and displayed by ESPript 3.0 [71].

### Identification of the orthologous *AAT* genes in sorghum, wheat, rice and *Arabidopsi*s, and their expression analysis

The orthologous *AAT* genes in sorghum, wheat, rice and *Arabidopsis* were identified as those in foxtail millet, and by OrthFinder2 with default parameters [72]. Their expression data in the main organs of root, stem, leaf, inflorescence/spike, and grain was obtained from the public transcriptome databases, respectively (wheat, http://www.wheat-expression.com; rice, http://expression.ic4r.org; sorghum, http://structuralbiology.cau.edu.cn/sorghum/;*Arabidopsis*, https://www.arabidopsis.org). Then, the conservation of the expression patterns of *AAT* orthologous genes among those species was investigated through the tissue-to-tissue and gene-to-gene correlation analysis, using the software of SPSS 19.0 (SPSS, Inc., Chicago, IL, USA) with Pearson approach.

To study the spatiotemporal expression patterns of *SiAATs* in various tissues and grain development, a merged transcriptome dataset of foxtail millet was constructed with the RNA-seq reads downloaded from Multi-omics Database for *Setaria italica* (MDSi, http://foxtail-millet.biocloud.net/home), including 13 tissues at the seedling, heading and filling stages, and 2 spikelets and 5 grains. The FPKM (Fragments Per Kilobase per Million) values were calculated using an R package Edge R [73]. The heat maps of log_2_ (FPKM+ 1) values were drawn by TBtools [67].

### Quality traits measurement and transcriptome analysis of two foxtail millet cultivars

Two foxtail millet cultivars “JG21” and “YG1” used in this experiment were widely grown in northern China and provided by Mr. Junjie Wang of Center for agricultural genetic resources research, shanxi agricultural university. They were grown in the experimental farm of the Institute of Water Saving Agriculture in Arid Areas of China, Northwest A&F University, Yangling, Shaanxi, China (34°7’N, 108°4’E) for two cropping seasons (May to September of 2017–2018 and 2018–2019), followed the local management practices. After harvest, the mature seeds were taken for measuring quality traits, including glutenin content, crude fiber content, crude fat content, and the contents of 18 essential amino acids of methionine, cysteine, phenylalanine, alanine, glycine, glutamic acid, arginine, lysine, tyrosine, leucine, proline, tryptophan, Serine, threonine, aspartic acid, valine, isoleucine, histidine, with a near-infrared analyzer DA7250 (Perten, Sweden).

The developing grains (include glumes) of “JG21” and “YG1” were separated at filling stage in 2019, and the total RNA was isolated using a Quick RNA Isolation kit (Takara Corporation, Dalian, China) [74], RNA purity and integrity was confirmed by an RNA Nano 6000 Assay Kit and the Bioanalyzer 2100 system (Agilent Technologies, CA, USA). The RNA-seq library construction and sequencing were performed with the 150 paired-end sequencing on Illumina HiSeq platform following the standard methods by Novogene Life Sciences Pvt. Ltd., Beijing, China with three biological replicates. After filtered the raw data, Clean reads were then mapped to the reference genome using Hisat2 [75]. Differentially expressed genes (DEGs) between “JG21” and “YG1” were identified using the R package DESeq [76], and the FPKM values were calculated [77]. The KEGG (Kyoto Encyclopedia of Genes and Genomes, http://www.genome.jp/kegg) and GO (Gene Ontology, http://geneontology.org/) annotation of DEGs were further performed with GOseq and KOBAS software, respectively [78]. All the analysis were performed following the standard methods.

### The expressions of some *AAT* genes in response to abiotic stresses

To verify the reliability of the transcriptome data from RNA-seq, the flag leaves, peduncles, roots, developing grains (include glumes) of “JG21” and developing grains (include glumes) of “YG1” were collected at the filling stage in 2019 for RNA extraction. For abiotic stresses, the raw read data of two datasets (SRA062640 and PRJNA545871) of foxtail millet under drought stress and salt stress were sourced from the NCBI SRA database to analyze the response of *SiAATs* to abiotic stresses [79, 80]. The detailed analysis process was described above.

The seedlings, 15 days after sowing (two leaves with a terminal bud) were exposed to 20% polyethylene glycol (PEG 6000) solution (Guangdong Guanghua Sci-Tech Co., Ltd., Guangdong, China) and NaCl (Xilong Scientific Co., Ltd., Guangdong, China) solution (200 mM) for 1 h and 5 h to mimic salt and drought stress, respectively, and all seedling plants were then collected for RNA isolation. PrimeScriptTM II 1st Strand cDNA Synthesis (Tiangen Biotech (Beijing) Co., Ltd., Beingjing, China) was used to synthesize the first strand of cDNA and Quantitative Real-time PCR (qRT-PCR) was conducted by SuperReal PreMix Color kit (SYBR Green) with the primers listed in Table 3 and Additional file 7: Table S3, with the *SiAct-7* gene as an internal reference. Each sample contained three biological replicates, and the 2^-ΔΔCT^ method was used for the statistical analysis.

## Supplementary Information

**Additional file 1: Figure S1.** Physical mapping and gene duplication of *SiAAT* genes in foxtail millet. Segmental duplication and tandem duplication are linked by red lines and blue shadows, respectively.

**Additional file 2: Figure S2.** The sequences of the 20 conserved motifs of SiAAT proteins. Different colors represent different amino acid residues. The larger the font is, the higher the proportion of this residue is as in the multiple sequence alignment.

**Additional file 3: Figure S3.** Gene structure of 25 paralogous *AAT* gene groups in foxtail millet. The gene structures of the tandemly and segmentally duplicated gene groups were on the left and the right respectively. Detail legend is as in Fig. 3.

**Additional file 4: Figure S4.** The expression levels of the 20 selected *SiAAT* genes in five main tissues at grain filling stage. The tissue names were listed in the X axis. The mean values of three replicates ± standard deviation (SD) were represented with Bars, and the *SiAct-7* gene is used as an internal reference.

**Additional file 5: Table S1.** The detail information and sequence characterization of 94 putative *AAT* genes in foxtail millet.

**Additional file 6: Table S2.** The orthologous *AAT* genes in foxtail millet, wheat, sorghum and *Arabidopsis*.

**Additional file 7: Table S3.** The primers used for the spatiotemporal expression analysis with Quantitative Real-Time PCR (qRT-PCR).

## Data Availability

The RNA-seq raw data of two foxtail millet cultivars “YG1” and “JG21” was submitted in the NCBI Sequence Read Archive (SRA) with the accession number PRJNA673778. The datasets supporting the conclusions of this article are included within the article and its additional files.

## Abbreviations

AAT: Amino acid transporter
APC: Amino acid-polyamine-choline
AAAP: Amino acid/auxin permease
ACT: Amino acid/choline transporter
CAT: Cationic amino acid transporter
PHS: Polyamine H^+^-symporter
TTP: Tyrosine-specific transporter
LHT: Lysine and histidine transporter
GAT: γ- aminobutyric acid transporter
ProT: Proline transporter
AAP: Amino acid permease
AUX: Auxin transporter
ANT: Aromatic and neutral amino acid transporter
GABA: γ-aminobutyric acid
QTL: Quantitative trait locus
pI: Isoelectric point
Mw: Molecular weight
TM: Transmembrane
qRT-PCR: Quantitative Real-Time PCR
GPC: Grain protein content
