# Supplementary figures and images for: Amino acid transporter (AAT) gene family in foxtail millet (Setaria italica L.): widespread family expansion, functional differentiation, roles in quality formation and response to abiotic stresses
Source: BMC Genomics. 2021 Jul 8;22:519. doi: 10.1186/s12864-021-07779-9 (PMC8268433; doi:10.1186/s12864-021-07779-9)

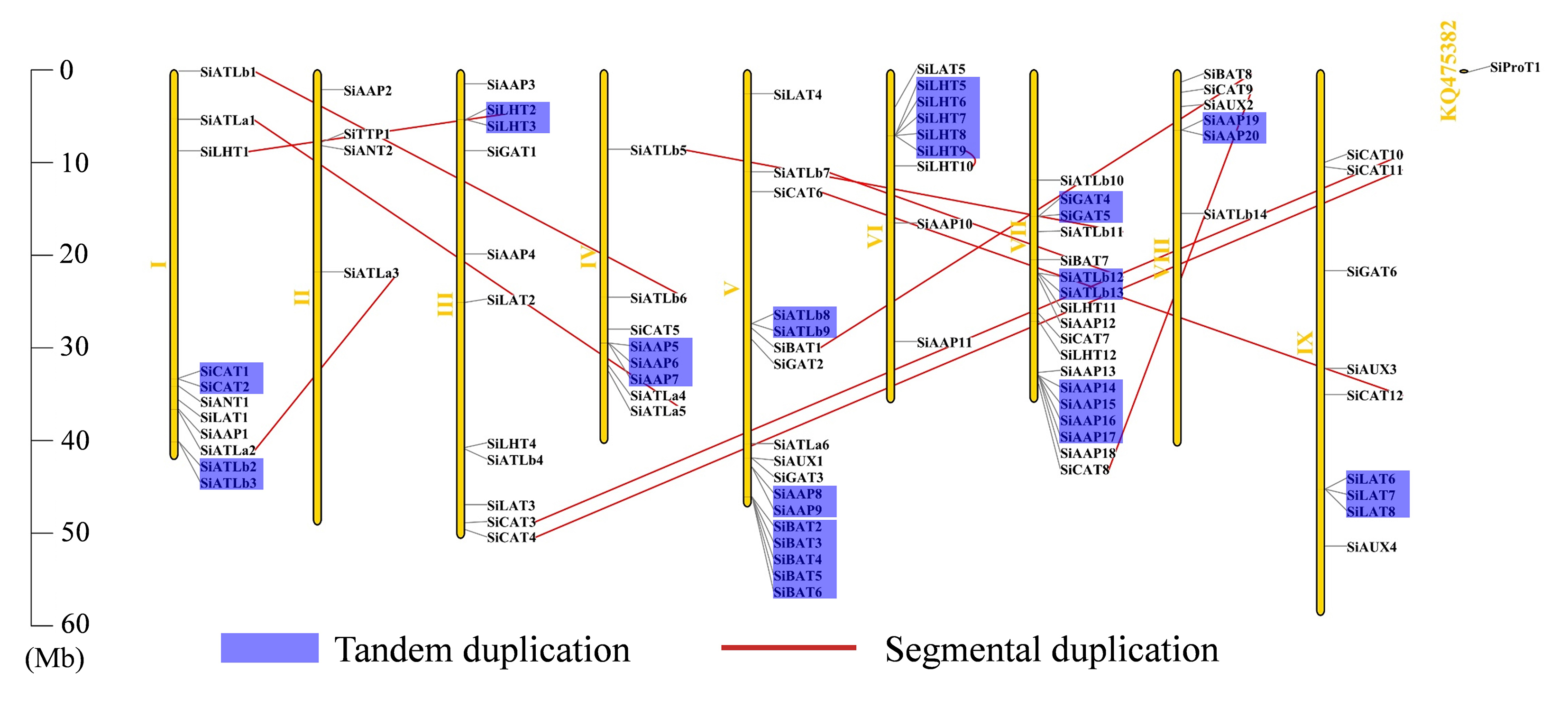

Supplement: Supplementary file 1 — Additional file 1: Figure S1. Physical mapping and gene duplication of SiAAT genes in foxtail millet. Segmental duplication and tandem duplication are linked by red lines and blue shadows, respectively. [file 12864_2021_7779_MOESM1_ESM.jpg]

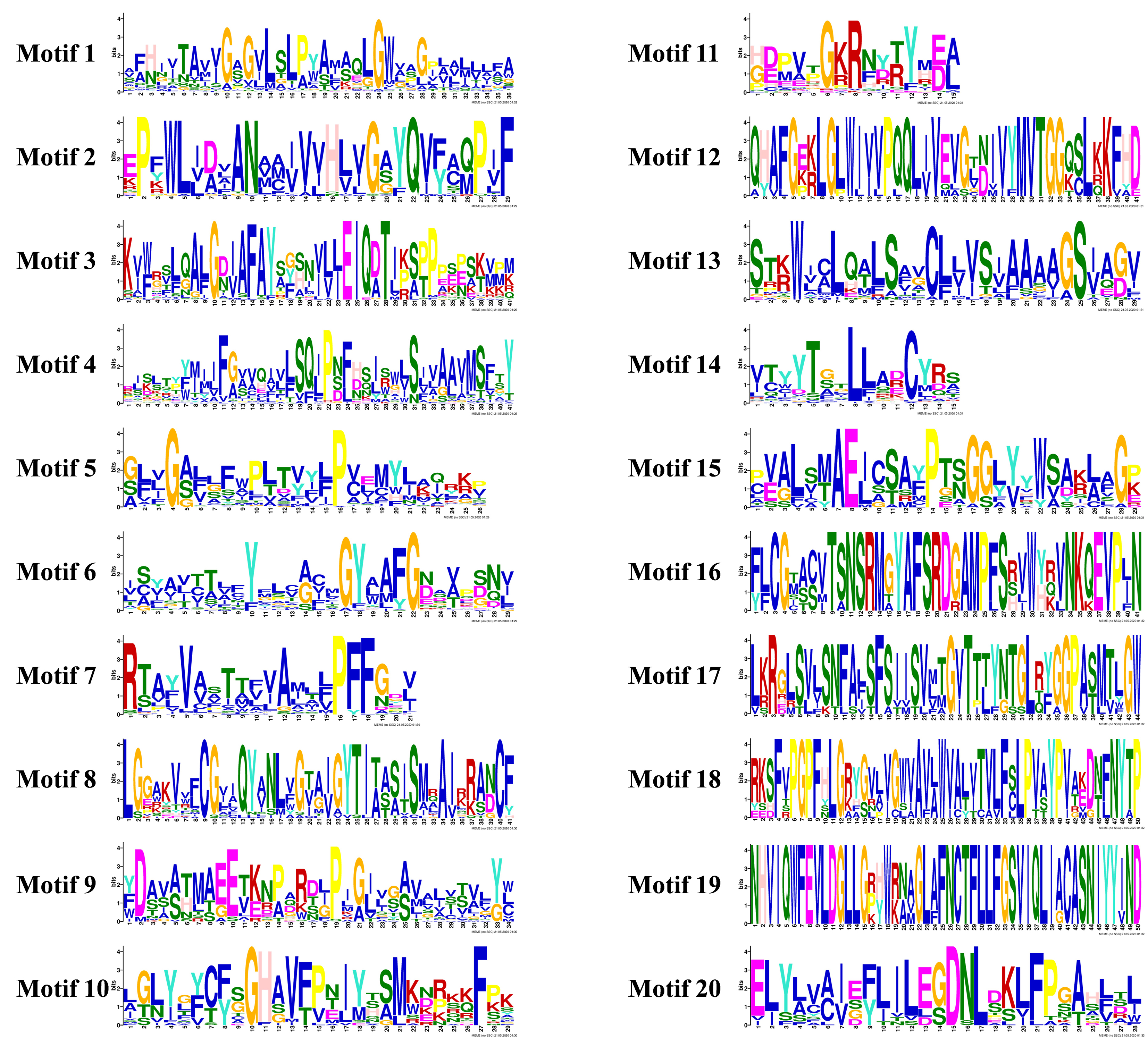

Supplement: Supplementary file 2 — Additional file 2: Figure S2. The sequences of the 20 conserved motifs of SiAAT proteins. Different colors represent different amino acid residues. The larger the font is, the higher the proportion of this residue is as in the multiple sequence alignment. [file 12864_2021_7779_MOESM2_ESM.jpg]

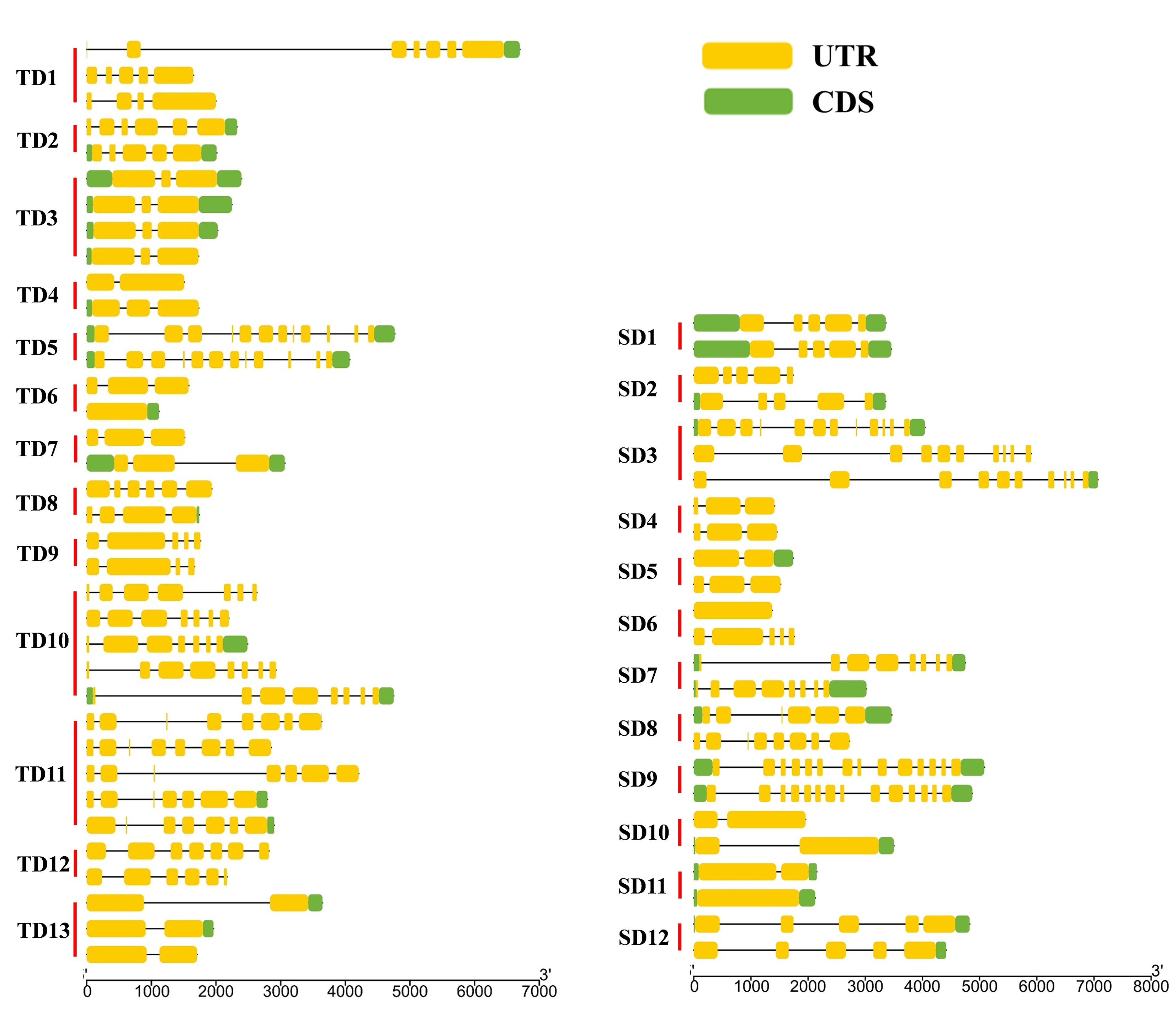

Supplement: Supplementary file 3 — Additional file 3: Figure S3. Gene structure of 25 paralogous AAT gene groups in foxtail millet. The gene structures of the tandemly and segmentally duplicated gene groups were on the left and the right respectively. Detail legend is as in Fig. 3. [file 12864_2021_7779_MOESM3_ESM.jpg]

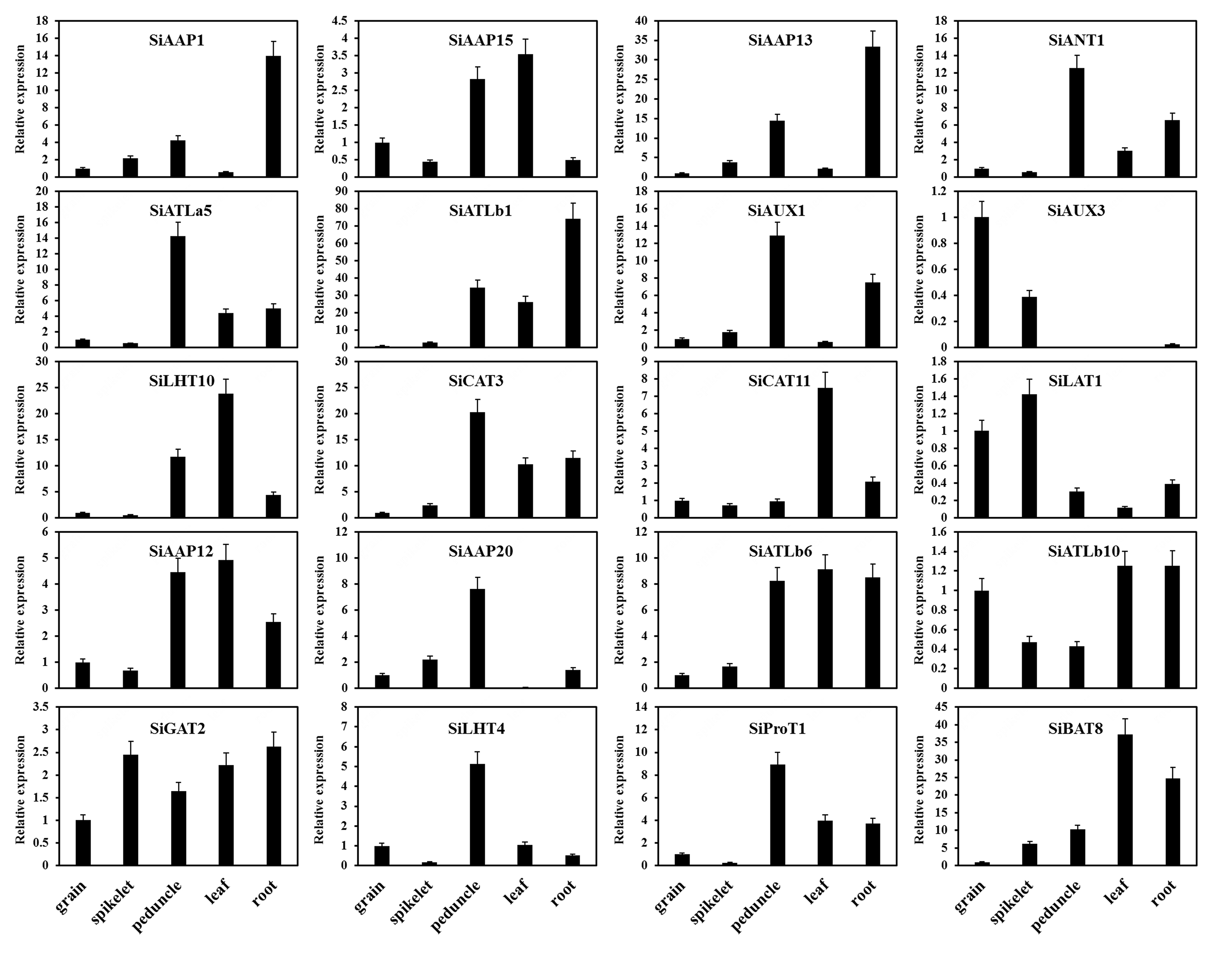

Supplement: Supplementary file 4 — Additional file 4: Figure S4. The expression levels of the 20 selected SiAAT genes in five main tissues at grain filling stage. The tissue names were listed in the X axis. The mean values of three replicates ± standard deviation (SD) were represented with Bars, and the SiAct-7 gene is used as an internal reference. [file 12864_2021_7779_MOESM4_ESM.jpg]
