# Supplementary material for: Amino acid transporter (AAT) gene family in foxtail millet (Setaria italica L.): widespread family expansion, functional differentiation, roles in quality formation and response to abiotic stresses
Source: BMC Genomics. 2021 Jul 8;22:519. doi: 10.1186/s12864-021-07779-9 (PMC8268433; doi:10.1186/s12864-021-07779-9)
Supplement: Supplementary file 5 — Additional file 5: Table S1. The detail information and sequence characterization of 94 putative AAT genes in foxtail millet. [file 12864_2021_7779_MOESM5_ESM.pdf]

Table S1. The detail information and sequence characterization of 94 putative *AAT* genes in foxtail millet.

No.	Gene[a]	Locus[b]	Gene Structure		ORF(bp)[e]	Protein[f]			TM region[g]	Subcellular Localization	Gene Duplication[h]
			Length(bp)[c]	Intron[d]		Size(aa)	MW(d)	pI			
	AAAP family										
	AAP group										
1	*SiAAP1*	SETIT_017136mg	5757	4	1392	463	49713.66	7.97	10	plasma membrane	
2	*SiAAP2*	SETIT_029675mg	4071	4	1452	483	52720.31	8.85	9	plasma membrane	
3	*SiAAP3*	SETIT_021976mg	2528	6	1395	464	50964.29	8.14	9	plasma membrane	
4	*SiAAP4*	SETIT_021862mg	2217	3	1485	494	53509.62	8.45	9	plasma membrane	
5	*SiAAP5*	SETIT_006252mg	6699	6	1551	516	56278.88	8.66	10	plasma membrane	TD1
6	*SiAAP6*	SETIT_007931mg	1651	4	1227	408	44684.85	6.58	8	plasma membrane	TD1
7	*SiAAP7*	SETIT_007877mg	1998	3	1389	462	50145.41	8.71	10	plasma membrane	TD1
8	*SiAAP8*	SETIT_001325mg	2326	5	1407	468	49948.22	9.04	10	plasma membrane	TD2
9	*SiAAP9*	SETIT_001577mg	2012	4	1272	423	45451.23	9.02	9	plasma membrane	TD2
10	*SiAAP10*	SETIT_013726mg	1787	3	1353	450	48383.75	9.38	9	plasma membrane	
11	*SiAAP11*	SETIT_015738mg	1422	1	1338	445	47209.15	9.57	9	plasma membrane	
12	*SiAAP12*	SETIT_011960mg	2249	5	1380	459	50741.99	8.4	9	plasma membrane	
13	*SiAAP13*	SETIT_010023mg	2637	3	1413	470	51104.66	8.46	8	plasma membrane	
14	*SiAAP14*	SETIT_009986mg	2393	2	1443	480	52147.41	7.02	9	plasma membrane	TD3
15	*SiAAP15*	SETIT_009998mg	2247	2	1434	477	51501.88	8.72	9	plasma membrane	TD3
16	*SiAAP16*	SETIT_009997mg	2027	2	1434	477	51502.73	8.27	9	plasma membrane	TD3
17	*SiAAP17*	SETIT_009983mg	1729	2	1446	481	51917.18	6.98	9	plasma membrane	TD3
18	*SiAAP18*	SETIT_012272mg	1421	2	1359	452	47524.59	9.1	7	plasma membrane	
19	*SiAAP19*	SETIT_027550mg	1509	1	1422	473	50880.06	8.61	10	plasma membrane	TD4
20	*SiAAP20*	SETIT_026316mg	1732	2	1422	473	50675.98	8.64	9	plasma membrane	TD4

No.	Gene[a]	Locus[b]	Gene Structure		ORF(bp)[e]	Protein[f]			TM region[g]	Subcellular Localization	Gene Duplication[h]
			Length(bp)[c]	Intron[d]		Size(aa)	MW(d)	pI			
	ANT group										
21	*SiANT1*	SETIT_017299mg	2192	0	1278	425	44847.08	6.74	11	vacuole membrane	
22	*SiANT2*	SETIT_030354mg	2692	2	1050	349	38178.87	6.3	9	vacuole membrane	
	ATLa group										
23	*SiATLa1*	SETIT_017155mg	3358	4	1377	458	49715.48	6.19	10	plasma membrane	SD1
24	*SiATLa2*	SETIT_019493mg	1736	4	1368	455	48693.09	8.11	11	plasma membrane	SD2
25	*SiATLa3*	SETIT_029801mg	3357	4	1368	455	49087.73	7.15	11	plasma membrane	SD2
26	*SiATLa4*	SETIT_008296mg	1469	1	1458	485	51414.72	6.32	10	vacuole membrane	
27	*SiATLa5*	SETIT_006392mg	3456	4	1380	459	49906.73	6.2	10	plasma membrane	SD1
28	*SiATLa6*	SETIT_001364mg	4921	3	1383	460	47991.38	9.21	11	vacuole membrane	
	ATLb group										
29	*SiATLb1*	SETIT_016787mg	4043	11	1698	565	61339.92	7.05	9	vacuole membrane	SD3
30	*SiATLb2*	SETIT_016828mg	4764	11	1647	548	59626.48	4.94	8	vacuole membrane	TD5
31	*SiATLb3*	SETIT_016920mg	4069	11	1557	518	55405.32	5.48	11	vacuole membrane	TD5
32	*SiATLb4*	SETIT_025256mg	3130	10	1755	584	62588.70	5.67	10	vacuole membrane	
33	*SiATLb5*	SETIT_008382mg	1411	2	1200	399	43335.64	9.24	11	vacuole membrane	SD4
34	*SiATLb6*	SETIT_008506mg	5901	6	1749	582	63487.07	8.08	8	vacuole membrane	SD3
35	*SiATLb7*	SETIT_001501mg	1739	1	1311	436	45394.60	7.96	11	vacuole membrane	SD5
36	*SiATLb8*	SETIT_004649mg	1577	2	1311	436	45589.27	8.04	10	vacuole membrane	TD6
37	*SiATLb9*	SETIT_0016082mg	1118	0	936	311	33069.36	8.9	9	vacuole membrane	TD6
38	*SiATLb10*	SETIT_009764mg	7068	9	1632	543	58575.60	6.31	9	vacuole membrane	SD3
39	*SiATLb11*	SETIT_012642mg	1454	2	1248	415	44083.37	9.52	11	vacuole membrane	SD4
40	*SiATLb12*	SETIT_012545mg	1518	2	1317	438	45709.13	8.54	9	vacuole membrane	TD7, SD5
41	*SiATLb13*	SETIT_010060mg	3065	2	1374	457	47538.01	8.56	10	vacuole membrane	TD7

No.	Gene[a]	Locus[b]	Gene Structure		ORF(bp)[e]	Protein[f]			TM region[g]	Subcellular Localization	Gene Duplication[h]
			Length(bp)[c]	Intron[d]		Size(aa)	MW(d)	pI			
42	*SiATLb14*	SETIT_028186mg	2389	1	1449	482	50810.81	9.23	10	vacuole membrane	
	AUX group										
43	*SiAUX1*	SETIT_004876mg	5472	6	1473	490	54623.93	8.49	10	plasma membrane	
44	*SiAUX2*	SETIT_026295mg	2227	4	1449	482	53350.06	8.83	10	plasma membrane	
45	*SiAUX3*	SETIT_035069mg	5030	3	1626	541	59701.37	9.1	10	plasma membrane	
46	*SiAUX4*	SETIT_035200mg	4391	6	1560	519	57729.28	8.9	10	plasma membrane	
	GAT group										
47	*SiGAT1*	SETIT_024570mg	2634	6	1446	481	51075.00	9.38	11	plasma membrane	
48	*SiGAT2*	SETIT_001355mg	5431	5	1386	461	49301.15	8.88	10	plasma membrane	
49	*SiGAT3*	SETIT_001363mg	5959	5	1383	460	49149.77	8.91	10	plasma membrane	
50	*SiGAT4*	SETIT_012737mg	1936	5	1410	469	49596.39	9.42	10	plasma membrane	TD8
51	*SiGAT5*	SETIT_010076mg	1740	3	1359	452	47915.42	9.66	10	plasma membrane	TD8
52	*SiGAT6*	SETIT_035729mg	3393	4	1329	442	47880.41	9.22	9	plasma membrane	
	LHT group										
53	*SiLHT1*	SETIT_019748mg	1370	0	1371	456	50249.23	9.23	10	plasma membrane	SD6
54	*SiLHT2*	SETIT_024807mg	1758	4	1347	448	49770.72	9.38	9	plasma membrane	TD9, SD6
55	*SiLHT3*	SETIT_025104mg	1668	3	1347	448	49262.31	9.28	10	plasma membrane	TD9
56	*SiLHT4*	SETIT_021791mg	3865	4	1539	512	55315.58	9.25	11	plasma membrane	
57	*SiLHT5*	SETIT_015309mg	2630	6	1290	430	48409.92	8.88	9	plasma membrane	TD10
58	*SiLHT6*	SETIT_014990mg	2198	6	1401	466	52689.09	9.22	10	plasma membrane	TD10
59	*SiLHT7*	SETIT_013742mg	2487	6	1338	445	49762.68	9.15	11	plasma membrane	TD10
60	*SiLHT8*	SETIT_015171mg	2926	7	1341	446	49761.85	9.06	10	plasma membrane	TD10
61	*SiLHT9*	SETIT_013740mg	4750	7	1341	446	49825.94	9.03	11	plasma membrane	TD10, SD7
62	*SiLHT10*	SETIT_013741mg	3022	7	1341	446	49606.56	9.19	10	plasma membrane	SD7

No.	Gene[a]	Locus[b]	Gene Structure		ORF(bp)[e]	Protein[f]			TM region[g]	Subcellular Localization	Gene Duplication[h]
			Length(bp)[c]	Intron[d]		Size(aa)	MW(d)	pI			
63	*SiLHT11*	SETIT_010148mg	2238	6	1305	434	47117.43	8.97	7	plasma membrane	
64	*SiLHT12*	SETIT_009824mg	2050	4	1578	525	56703.08	9.61	11	plasma membrane	
	ProT group										
65	*SiProT1*	SETIT_020692mg	3347	7	1401	466	51178.73	8.55	11	plasma membrane	
	APC family										
	ACT group										
66	*SiBAT1*	SETIT_000996mg	3462	5	1581	526	56405.59	9.05	12	vacuole membrane	SD8
67	*SiBAT2*	SETIT_001031mg	3634	7	1566	521	55786.10	8.41	12	vacuole membrane	TD11
68	*SiBAT3*	SETIT_003988mg	2852	7	1524	507	54651.71	8.7	12	vacuole membrane	TD11
69	*SiBAT4*	SETIT_001029mg	4206	6	1566	521	55693.89	9.06	12	vacuole membrane	TD11
70	*SiBAT5*	SETIT_001036mg	2797	6	1563	520	55770.79	8.71	12	vacuole membrane	TD11
71	*SiBAT6*	SETIT_000982mg	2900	6	1596	531	56912.14	8.87	12	vacuole membrane	TD11
72	*SiBAT7*	SETIT_009827mg	3898	5	1572	523	57201.82	7.97	13	vacuole membrane	
73	*SiBAT8*	SETIT_026229mg	2725	7	1560	519	56007.10	8.99	12	vacuole membrane	SD8
	CAT group										
74	*SiCAT1*	SETIT_016730mg	2819	6	1677	558	59378.15	8.63	12	chloroplast membrane	TD12
75	*SiCAT2*	SETIT_016768mg	2169	5	1290	429	45515.50	6.11	9	chloroplast membrane	TD12
76	*SiCAT3*	SETIT_021473mg	5083	13	1878	625	66573.78	5.75	14	vacuole membrane	SD9
77	*SiCAT4*	SETIT_025200mg	1956	1	1791	596	62832.04	6.71	14	plasma membrane	SD10
78	*SiCAT5*	SETIT_006108mg	1930	0	1800	599	62494.41	8.71	14	plasma membrane	
79	*SiCAT6*	SETIT_000719mg	2151	1	1842	613	65645.24	8.56	13	plasma membrane	SD11
80	*SiCAT7*	SETIT_009732mg	3162	3	1683	560	60936.40	8.74	13	plasma membrane	
81	*SiCAT8*	SETIT_009648mg	4826	4	1800	599	62968.54	8.86	14	plasma membrane	SD12
82	*SiCAT9*	SETIT_026149mg	4413	4	1779	592	61691.77	8.88	14	plasma membrane	SD12

No.	Gene[a]	Locus[b]	Gene Structure		ORF(bp)[e]	Protein[f]			TM region[g]	Subcellular Localization	Gene Duplication[h]
			Length(bp)[c]	Intron[d]		Size(aa)	MW(d)	pI			
83	*SiCAT10*	SETIT_034651mg	4874	13	1908	635	67481.97	5.45	14	vacuole membrane	SD9
84	*SiCAT11*	SETIT_034783mg	3499	1	1809	602	64439.87	7.57	14	plasma membrane	SD10
85	*SiCAT12*	SETIT_034804mg	2124	0	1785	594	63078.63	9.36	11	plasma membrane	SD11
	PHS group										
86	*SiLAT1*	SETIT_016864mg	1778	0	1443	480	51577.77	5.25	10	plasma membrane	
87	*SiLAT2*	SETIT_021459mg	3721	4	1899	632	71036.95	6.25	7	plasma membrane	
88	*SiLAT3*	SETIT_021637mg	2148	2	1683	560	59655.20	7.71	9	chloroplast membrane	
89	*SiLAT4*	SETIT_004154mg	9418	12	2955	984	107547.32	6.66	11	plasma membrane	
90	*SiLAT5*	SETIT_013633mg	3494	1	1482	493	54350.13	6.02	12	plasma membrane	
91	*SiLAT6*	SETIT_035377mg	3646	1	1476	491	51498.78	8.76	9	vacuole membrane	TD13
92	*SiLAT7*	SETIT_035309mg	1960	1	1512	503	51439.63	8.53	10	plasma membrane	TD13
93	*SiLAT8*	SETIT_035306mg	1707	1	1512	503	53748.41	9.08	9	vacuole membrane	TD13
	TTP group										
94	*SiTTP1*	SETIT_029599mg	2389	1	1497	498	52366.62	9.99	11	vacuole membrane	

[a] Systematic designation given to foxtail millet *AATs* in this study. [b] Locus identity number of *SiAATs* in foxtail millet genome. [c] Gene full length of *SiAATs*. [d] Number of introns in *SiAAT* genes. [e] Length of the open reading frame for *SiAATs*. [f] Protein characterization of SiAATs. [g] Number of transmembrane regions of SiAATs, predicted by the TMHMM Server v2.0. [h] The duplicated genes, TD and SD represent tandem duplication and segmental duplication, respectively. The following numbers represent different pairs of duplicated genes.
