# Supplementary material for: Amino acid transporter (AAT) gene family in foxtail millet (Setaria italica L.): widespread family expansion, functional differentiation, roles in quality formation and response to abiotic stresses
Source: BMC Genomics. 2021 Jul 8;22:519. doi: 10.1186/s12864-021-07779-9 (PMC8268433; doi:10.1186/s12864-021-07779-9)
Supplement: Supplementary file 6 — Additional file 6: Table S2. The orthologous AAT genes in foxtail millet, wheat, sorghum and Arabidopsis. [file 12864_2021_7779_MOESM6_ESM.pdf]

Table S2. The orthologous *AAT* genes in foxtail millet, wheat, sorghum and *Arabidopsis*.

Foxtail millet[a]		Sorghum	Wheat				Rice		*Arabidopsis*	
**Gene ID**	**Locus**		**Gene ID**	**A**	**B**	**D**	**Gene ID**	**Locus**	**Gene ID**	**Locus**
SiAAP1	SETIT_017136mg	SORBI_3004G256800	TaAAP17	TraesCS6A02G285300	TraesCS6B02G314200	TraesCS6D02G265800	OsAAP10	Os02g0722400	AtAAP7	AT5G23810
SiAAP2	SETIT_029675mg	SORBI_3002G027500	TaAAP2	TraesCS2A02G268200	TraesCS2B02G266600	TraesCS2D02G256000	OsAAP1	Os07g0134000	NA	NA
SiAAP3	SETIT_021976mg	SORBI_3006G250200	TaAAP6	TraesCS2A02G499800	TraesCS2B02G527800	TraesCS2D02G500000	OsAAP14	Os04g0659800	AtAAP2	AT5G09220
SiAAP4	SETIT_021862mg	SORBI_3009G142800	TaAAP1	TraesCS1A02G264500	TraesCS1B02G275200	TraesCS1D02G264700	OsAAP7	Os05g0424000	AtAAP3	AT1G77380
SiAAP5	SETIT_006252mg	SORBI_3010G166500	TaAAP22	TraesCS7A02G356639	TraesCS7B02G271151	TraesCS7D02G366000	OsAAP3	Os06g0556000	AtAAP1	AT1G58360
SiAAP8	SETIT_001325mg	SORBI_3003G375900	TaAAP9	TraesCS3A02G388100	TraesCS3B02G420600	TraesCS3D02G381400	OsAAP5	Os01g0878400	AtAAP8	AT1G10010
SiAAP12	SETIT_011960mg	SORBI_3006G115300	TaAAP3	TraesCS2A02G331100	TraesCS2B02G351100	TraesCS2D02G331800	OsAAP13	Os04g0470700	NA	NA
SiAAP13	SETIT_010023mg	SORBI_3008G068200	TaAAP15	TraesCS5A02G115800	TraesCS5B02G120800	TraesCSU02G134900	OsAAP4	Os12g0194900	AtAAP6	AT5G49630
SiAAP14	SETIT_009986mg	SORBI_3008G058900	TaAAP14	TraesCS5A02G115100	TraesCS5B02G116100	TraesCS5D02G125700	NA	NA	NA	NA
SiAAP18	SETIT_012272mg	SORBI_3008G057200	TaAAP13	TraesCS5A02G113300	TraesCS5B02G120000	TraesCS5D02G129000	NA	NA	NA	NA
SiAAP19	SETIT_027550mg	SORBI_3005G072300	NA	NA	NA	NA	NA	NA	NA	NA
SiANT1	SETIT_017299mg	SORBI_3004G285800	TaANT6	TraesCS6A02G254100	TraesCS6B02G271500	TraesCS6D02G235500	NA	NA	AtANT4	AT2G42005
SiANT2	SETIT_030354mg	SORBI_3002G082900	TaANT4	TraesCS5A02G472500	TraesCS5B02G485400	TraesCS5D02G485100	NA	NA	AtANT1	AT3G11900
SiATLa1	SETIT_017155mg	SORBI_3004G074500	TaATLa2	TraesCS6A02G170700	TraesCS6B02G198900	TraesCS6D02G160400	OsATL6	Os02g0191300	NA	NA
SiATLa5	SETIT_006392mg	SORBI_3010G199200	TaATLa5	TraesCS7A02G391600	TraesCS7B02G293500	TraesCS7D02G387200	OsATL5	Os06g0633800	AtT3	AT3G30390
SiATLa2	SETIT_019493mg	SORBI_3004G253200	TaATLa3	TraesCS6A02G288200	TraesCS6B02G317700	TraesCS6D02G270800	OsATL3	Os02g0727100	NA	NA
SiATLa4	SETIT_008296mg	SORBI_3010G206000	TaATLa6	TraesCS7A02G517100	TraesCS7B02G433400	TraesCS7D02G507300	OsATL1	Os06g0644700	AtT5	AT1G80510
SiATLa6	SETIT_001364mg	SORBI_3003G340800	TaATLa1	TraesCS3A02G346700	TraesCS3B02G378500	TraesCS3D02G340400	OsATL7	Os01g0825800	AtT1	AT2G40420
SiATLb1	SETIT_016787mg	SORBI_3004G001000	TaATLb11	TraesCS6A02G002400	TraesCS6B02G007400	TraesCS6D02G003200	OsATL11	Os02g0101000	AtVAAT4	AT2G39130
SiATLb2	SETIT_016828mg	SORBI_3004G325100	TaATLb12	TraesCS6A02G348300	TraesCS6B02G381900	TraesCS6D02G331300	OsATL9	Os02g0788800	AtVAAT3	AT2G41190
SiATLb4	SETIT_025256mg	SORBI_3008G139500	TaATLb10	TraesCS5A02G081500	TraesCS5B02G084900	TraesCS5D02G091100	OsATL10	Os12g0574000	NA	NA
SiATLb5	SETIT_008382mg	SORBI_3010G092700	TaATLb14	TraesCS7A02G188100	TraesCS7B02G093100	TraesCS7D02G189100	OsATL12	Os06g0228500	NA	NA

Foxtail millet[a]		Sorghum	Wheat				Rice		*Arabidopsis*	
Gene ID	Locus		Gene ID	A	B	D	Gene ID	Locus	Gene ID	Locus
SiATLb6	SETIT_008506mg	SORBI_3010G146700	NA	NA	NA	NA	NA	NA	NA	NA
SiATLb8	SETIT_004649mg	SORBI_3003G207900	NA	NA	NA	NA	OsATL16	Os01g0597400	AtVAAT2	AT5G15240
SiATLb10	SETIT_009764mg	SORBI_3006G024600	TaATLb2	TraesCS2A02G052500	TraesCS2B02G065200	TraesCSU02G023100	New 1	Os04g0201800	NA	NA
SiATLb12	SETIT_012545mg	SORBI_3006G106700	TaATLb4	TraesCS2A02G338600	TraesCS2B02G343300	TraesCS2D02G324400	OsATL13	Os04g0460300	AtVAAT1	AT3G28960
SiAUX1	SETIT_004876mg	SORBI_3003G361300	TaAUX3	TraesCS3A02G369200	TraesCS3B02G401000	TraesCS3D02G362100	OsAUX1	Os01g0856500	AtAUX1	AT2G38120
SiAUX2	SETIT_026295mg	SORBI_3005G052500	TaAUX5	TraesCS4A02G207600	TraesCS4B02G111100	TraesCS4D02G108800	OsAUX5	Os11g0169200	AtLAX3	AT1G77690
SiAUX3	SETIT_035069mg	SORBI_3001G267100	TaAUX1	TraesCS1A02G077800	TraesCS1B02G095900	TraesCS1D02G079900	OsAUX3	Os03g0244600	AtLAX2	AT2G21050
SiAUX4	SETIT_035200mg	SORBI_3001G439000	TaAUX4	TraesCS4A02G070500	TraesCS4B02G226500	TraesCS4D02G227200	OsAUX4	Os10g0147400	NA	NA
SiGAT1	SETIT_024570mg	SORBI_3009G250200	TaGAT4	TraesCS3A02G224100	TraesCS3B02G253600	TraesCS3D02G229500	OsGAT1	Os05g0586500	NA	NA
SiGAT2	SETIT_001355mg	SORBI_3003G223800	TaGAT1	TraesCS1A02G425300	TraesCS1B02G459700	TraesCS1D02G434300	OsGAT2	Os01g0621200	AtGATL1	AT5G41800
SiGAT3	SETIT_001363mg	SORBI_3003G362100	TaGAT5	TraesCS3A02G370300	TraesCS3B02G402200	TraesCS3D02G363200	OsGAT3	Os01g0857400	AtGAT1	AT1G08230
SiGAT6	SETIT_035729mg	SORBI_3001G249000	TaGAT3	TraesCS3A02G102300	TraesCS3B02G120100	TraesCS3D02G104600	OsGAT4	Os10g0415100	NA	NA
SiLHT2	SETIT_024807mg	SORBI_3009G087900	NA	NA	NA	NA	NA	NA	NA	NA
SiLHT4	SETIT_021791mg	SORBI_3008G097700	NA	NA	NA	NA	OsLHT6	Os12g0485600	NA	NA
SiLHT5	SETIT_015309mg	SORBI_3007G025700	NA	NA	NA	NA	NA	NA	NA	NA
SiLHT8	SETIT_015171mg	SORBI_3007G025600	NA	NA	NA	NA	NA	NA	NA	NA
SiLHT10	SETIT_013741mg	SORBI_3007G092400	NA	NA	NA	NA	NA	NA	NA	NA
SiLHT11	SETIT_010148mg	SORBI_3006G108300	TaLHT2	TraesCS2A02G337400	TraesCS2B02G344500	TraesCS2D02G325500	NA	NA	AtLHT9	AT1G25530
SiLHT12	SETIT_009824mg	SORBI_3006G178500	TaLHT3	TraesCS2A02G406000	TraesCS2B02G423700	TraesCS2D02G402900	NA	NA	AtLHT4	AT1G47670
SiProT1	SETIT_020692mg	SORBI_3002G001000	TaProT1	TraesCS2A02G268400	TraesCS2B02G287200	TraesCS2D02G267400	OsProT3	Os07g0100800	NA	NA
SiBAT2	SETIT_001031mg	SORBI_3003G422400	TaBAT4	TraesCS3A02G484600	TraesCS3B02G531700	TraesCS3D02G480100	OsBAT2	Os01g0945100	NA	NA
SiBAT7	SETIT_009827mg	SORBI_3006G084600	TaBAT1	TraesCS2A02G305900	TraesCS2B02G322600	TraesCS2D02G304300	OsBAT7	Os04g0435100	AtBAT1	AT2G01170
SiBAT8	SETIT_026229mg	SORBI_3008G015300	NA	NA	NA	NA	NA	NA	NA	NA
SiCAT1	SETIT_016730mg	SORBI_3004G296300	TaCAT11	TraesCS6A02G244400	TraesCS6B02G280000	TraesCS6D02G226700	OsCAT2	Os02g0655700	AtCAT9	AT1G05940

Foxtail millet[a]		Sorghum	Wheat				Rice		*Arabidopsis*	
**Gene ID**	**Locus**		**Gene ID**	**A**	**B**	**D**	**Gene ID**	**Locus**	**Gene ID**	**Locus**
SiCAT3	SETIT_021473mg	SORBI_3008G177200	TaCAT6	TraesCS5A02G025400	TraesCS5B02G023300	TraesCS5D02G031800	OsCAT11	Os12g0623500	AtCAT2	AT1G58030
SiCAT4	SETIT_025200mg	SORBI_3008G168500	TaCAT7	TraesCS5A02G042900	TraesCS5B02G046500	TraesCS5D02G051400	OsCAT10	Os12g0613100	AtCAT1	AT4G21120
SiCAT5	SETIT_006108mg	SORBI_3010G159400	NA	NA	NA	NA	OsCAT6	Os06g0539400	AtCAT8	AT1G17120
SiCAT6	SETIT_000719mg	SORBI_3003G022600	TaCAT4	TraesCS3A02G133100	TraesCS3B02G166500	TraesCS3D02G149300	OsCAT1	Os01g0209800	AtCAT5	AT2G34960
SiCAT7	SETIT_009732mg	SORBI_3006G165200	TaCAT2	TraesCS2A02G390100	TraesCS2B02G409100	TraesCS2D02G388600	OsCAT5	Os04g0543600	AtCAT7	AT3G10600
SiCAT8	SETIT_009648mg	SORBI_3008G040900	NA	NA	NA	NA	OsCAT8	Os11g0155500	NA	NA
SiCAT9	SETIT_026149mg	SORBI_3005G041300	TaCAT8	TraesCS5A02G126900	TraesCS5B02G126000	TraesCS5D02G134500	OsCAT9	Os12g0156866	NA	NA
SiCAT10	SETIT_034651mg	SORBI_3001G151000	NA	NA	NA	NA	OsCAT4	Os03g0654400	AtCAT2	AT1G58030
SiCAT11	SETIT_034783mg	SORBI_3001G157200	TaCAT10	TraesCS5A02G375600		TraesCS5D02G385125	OsCAT10	Os12g0613100	NA	NA
SiLAT1	SETIT_016864mg	SORBI_3004G270400	TaLAT9	TraesCS6A02G269200	TraesCS6B02G296500	TraesCS6D02G245700	OsLAT1	Os02g0700500	AtLAT3	AT1G31830
SiLAT3	SETIT_021637mg	SORBI_3008G142600	TaLAT6	TraesCS5A02G073000	TraesCS5B02G079100	TraesCS5D02G085800	OsLAT7	Os12g0580400	AtLAT4	AT3G19553
SiLAT4	SETIT_004154mg	SORBI_3009G105900	NA	NA	NA	NA	OsLAT8	Os01g0304100	NA	NA
SiLAT5	SETIT_013633mg	SORBI_3007G053100	NA	NA	NA	NA	NA	NA	NA	NA
SiLAT6	SETIT_035377mg	SORBI_3001G357500	NA	NA	NA	NA	OsLAT4	Os03g0375966	AtLAT5	AT3G13620
SiLAT7	SETIT_035309mg	SORBI_3001G357600	NA	NA	NA	NA	NA	NA	NA	NA
SiTTP1	SETIT_029599mg	SORBI_3002G079900	TaTTP2	TraesCS4A02G055300	TraesCS4B02G249300	TraesCS4D02G249600	New 2	Os07g0225100	AtTTP1	AT2G33260

[a] The genome sequences of all species are downloaded from Ensemble plants database (**http://plants.ensembl.org/info/website/ftp/index.html**).
