# Supplementary material for: Amino acid transporter (AAT) gene family in foxtail millet (Setaria italica L.): widespread family expansion, functional differentiation, roles in quality formation and response to abiotic stresses
Source: BMC Genomics. 2021 Jul 8;22:519. doi: 10.1186/s12864-021-07779-9 (PMC8268433; doi:10.1186/s12864-021-07779-9)
Supplement: Supplementary file 7 — Additional file 7: Table S3. The primers used for the spatiotemporal expression analysis with Quantitative Real-Time PCR (qRT-PCR). [file 12864_2021_7779_MOESM7_ESM.pdf]

Table S3. The primers used for the spatiotemporal expression analysis with Quantitative Real-Time PCR (qRT-PCR).

**Genes**	**Locus ID**	**Forward primers(5'→3')**	**Reverse primers(5'→3')**	**Amplicon size (bp)**
SiAct-7	SETIT_0396062mg	CAGGGAGAAGATGACCCAAATC	CACCAGAGTCCAGCACAATAC	125
SiAAP1	SETIT_017136mg	CTCATCGCCGACTGCTACAT	ACAGATGGCTCCTCTCACCT	106
SiAAP15	SETIT_009998mg	CTTAGCCTCTTCCGCCTGAC	CGACTGGGAAGTAGACGGTG	142
SiAAP13	SETIT_010023mg	ACCTACATGGTGGTGTTCGG	GGCTGATGAAGGAGTAGCCG	121
SiANT1	SETIT_017299mg	TTTCAGGTACGTTGGTCGGG	TGGAACAATGCGACGATGGA	134
SiATLa5	SETIT_006392mg	TGACCATGATCGTGCTTGCT	TGCATCGACGACAGTTCTCC	124
SiATLb1	SETIT_016787mg	GGACCCCAACTCTAAGCAGG	GCTCTTTCGCGCTTCTTCAC	143
SiAUX1	SETIT_004876mg	CCGTCTACATCATCCCGTCG	TCAACACGAACATCCCCGTC	120
SiAUX3	SETIT_035069mg	CTGGACCTACATCTTCGGCG	CGATGTACCAGGCGGTGTAG	119
SiLHT10	SETIT_013741mg	CGACTACGACACCAGGGAAG	TCGAAGGGATGGTAGCCTGA	111
SiCAT3	SETIT_021473mg	GTACGCAAGCAAGCTGGATG	GGCTGAAGTTGTGAGGACCA	97
SiCAT11	SETIT_034783mg	AACCACAAGCCCAACGACTT	GGACGATGGAGAGCACGTAG	160
SiLAT1	SETIT_016864mg	GTTCTTCGTCATGGGGCTCA	ACAGCGTGTTGAGGTACAGG	104
SiAAP12	SETIT_011960mg	CTACCTCGGCAAGAAGCACA	GGCGGTGGTGATGGTATAGG	91
SiAAP20	SETIT_026316mg	GGCAAGCGGAACTACACCTA	GATGGTGTAACCGATGGCGA	120
SiATLb6	SETIT_008506mg	AAGCAGGCGCAAATTAACGG	CGGAGCTCTCGATGATGAGG	116
SiATLb10	SETIT_009764mg	ACCTACCCAGACATCGGACA	GTGCGTTGGGGAACAACTTT	121
SiGAT2	SETIT_001355mg	TCGCTATTGACTCGCCCATC	GAGAGAGAGACAGGCACACG	111
SiLHT4	SETIT_021791mg	CCAGGCAGGAGGTACAACAG	CCCCTCCAACCAGGATCAAC	136
SiProT1	SETIT_020692mg	CCATAAGGCCTCCTGTGGTC	ACTTGATGTCGCGGAACCAT	125
